# Micropatterned DNA Hydrogels for Spatiotemporal Programming of Chemical Reaction Networks

**DOI:** 10.1021/acsnano.5c20505

**Published:** 2026-03-14

**Authors:** Kohei Nishiyama, Piet J. M. Swinkels, Brigitta Dúzs, Weixiang Chen, Andreas Walther

**Affiliations:** Life-like Materials and Systems, Department of Chemistry,University of Mainz, Duesbergweg 10-14, 55128 Mainz, Germany

**Keywords:** DNA nanoscience, strand displacement reaction, reaction-diffusion system, 3D printing, cellular communication

## Abstract

Cells in living systems communicate by sending and receiving signal molecules to coordinate their behavior. To achieve long-distance and noise-resistant communication, cells pattern themselves into spatially organized structures. Inspired by this strategy, systems biology and materials science have aimed to construct artificial communication systems whose dynamics can be controlled by their spatial arrangement. However, experimental understanding of how spatial arrangement influences communication remains limited, mainly due to the difficulty of precisely positioning multiple artificial cellular agents. Here, we demonstrate that communication between artificial cell-like units can be programmed using DNA-based reaction networks and tuned by their spatial arrangement. Arrays of DNA-functionalized hydrogel posts were fabricated as artificial cellular units using a microscale 3D printing technique, enabling precise and flexible control over their geometry and arrangement. The communication between the posts and the collective behavior of the array can be rationally programmed by implementing DNA-based chemical reaction networks. The arrays can sense locally added DNA stimuli and exhibit transient activation patterns that are unique to input positions. This concept is further extended to post-to-post communication, where catalytic signal amplification in a spatially separated configuration leads to spatially biased activation. Finally, by introducing a negative feedback loop between two post types, we achieve more complex spatiotemporal dynamics in which the collective behavior is strongly influenced by spatial arrangement. Our system provides a simple yet versatile experimental platform for exploring arrangement-governed communication among artificial cellular agents and offers insights into the design of functional systems empowered by collective chemical intelligence.

## Graphical abstract

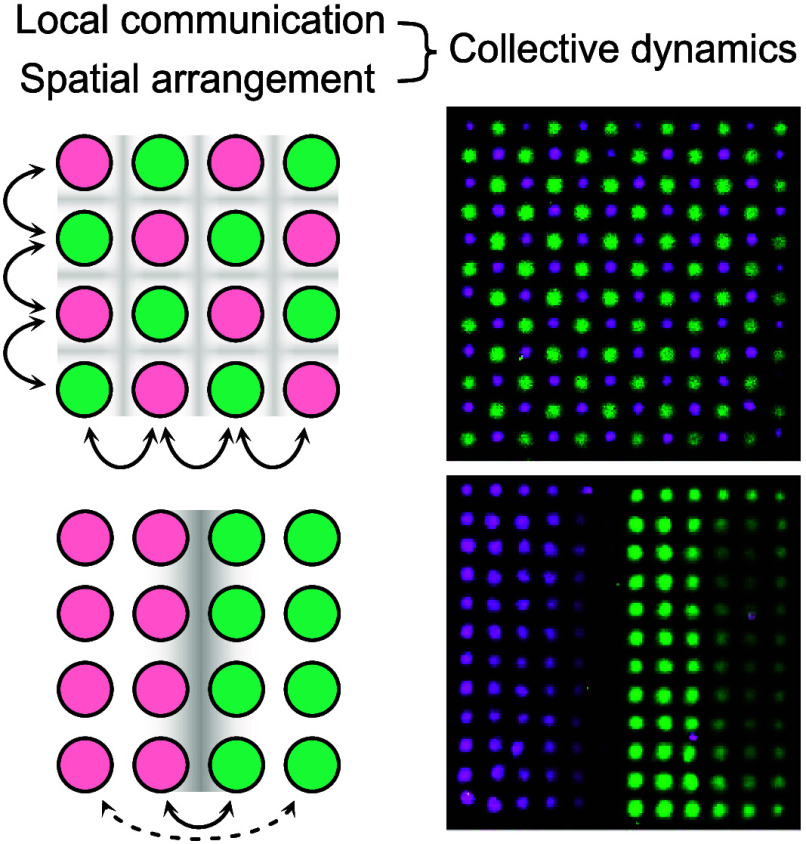

In living systems, cells communicate with one another by transmitting diffusible signal molecules to their neighbors.^1^ This molecular communication facilitates collective information processing, enabling cells to coordinate their activities and achieve complex, spatiotemporal regulation of their functions. Examples range from quorum sensing in bacterial colonies, which regulates gene expression based on population density,^2^ to the intricate processes of morphogenesis that generate complex patterns through the mutual regulation of neighboring cells.^3^ A grand challenge in synthetic biology and materials science is to construct artificial molecular systems that mimic such multicellular communication to provide autonomous functionality with embodied intelligence.^4,5^

Intercellular communication is governed by multiple factors, including chemical reactions inside and outside the cells, cell density, signal diffusivity, and, particularly, their spatial arrangement.^6^ Diffusible signal molecules are efficiently transmitted only over short distances, typically up to a few hundred micrometers.^7^ Beyond that distance, the signal becomes masked by background noise.^8^ To achieve long-distance communication under this constraint, biological systems repetitively pattern themselves into small functional units.^9^ For example, the liver divides its internal cells into hexagonal repeating units called lobules. Within these lobules, cells with different functions are arranged in a spatially organized manner, allowing for efficient communication among cells irrespective of the overall size of the animal.^9^ This strategy can also be useful for constructing artificial materials and systems, allowing for rationally tuning the communicability of artificial cells via their precise arrangement.

Inspired by these biological principles, recent research has focused on constructing artificial systems that mimic intercellular communication. A functional artificial cell requires two essential components: the ability to receive, process, and transmit signals, and compartmentalization to define its boundary. For signal processing, chemical reaction networks based on DNA circuits,^10^ transcriptional circuits,^11–13^ and enzymatic cascades^14–17^ are widely used due to their rational designability. Various compartmentalization strategies have also been proposed, including membrane-based structures such as liposomes,^18,19^ water-in-oil droplets,^12,13,20^ polymer capsules,^21^ and proteinosomes,^22–24^ and membrane-less structures like coacervates,^25–27^ hydrogels,^28–31^ and surface-deposited systems such as grafted colloids^32^ and interconnected microchambers.^33,34^ However, despite the well-known importance of spatial organization *in vivo*, it remains poorly understood how cell positioning and signal localization affect communication in artificial systems. This limitation arises because artificial cells are typically positioned stochastically, and robust experimental platforms that enable the free placement of multiple communicating units at arbitrary locations while preserving well-defined chemical functionality remain difficult to access.

Micropatterned hydrogels provide an ideal platform for constructing spatiotemporally organized artificial cellular systems to address this challenge. By conjugating DNA to hydrogel networks, they can serve as scaffolds for various DNA-based reactions.^31,35–37^ Furthermore, recent advances in microscale 3D printing techniques enable the maskless high-resolution patterning of such hydrogels on substrates.^38–41^ This technique enables precise control over the size, shape, and spatial arrangement of functionalized hydrogel units, making it ideal for studying communication between them. Several studies have successfully demonstrated DNA-based signal propagation and unidirectional communication among patterned hydrogels.^29,30,42,43^ However, how the spatial arrangement and local communication among hydrogels determine their collective spatiotemporal behavior remains largely unexplored.

Herein, we demonstrate that the collective spatiotemporal behavior of micropatterned artificial cell-like compartments can be programmed through local communication. Using a microprinting technique, we create arrays of DNA-functionalized hydrogel posts as a platform to investigate the communication. The populations and the arrangements of the hydrogel posts can be freely designed by altering the UV illumination patterns, providing a high degree of flexibility and control over the system architecture. First, we show that arrays composed of a single type of hydrogel post can sense locally added activation and inhibition signals, generating transient spatiotemporal patterns. Next, we extend this system to arrays composed of two distinct types of posts and demonstrate that one type of post can locally activate the other through direct signal transmission by implementing catalytic signal amplification. Finally, we implement more complex communication between the two types of posts regulated by negative feedback. We demonstrate that the spatial arrangement of posts can drastically tune the degree of local and global activation across the array. Our system reveals how geometry and spatial organization can be harnessed to engineer artificial systems that emulate multicellular communication.

## Results and Discussion

### Fabrication of DNA-Functionalized Hydrogel Post Arrays

We fabricated arrays of DNA-functionalized hydrogel posts on glass surfaces within polydimethylsiloxane (PDMS)-based microfluidic channels using microscale continuous optical printing (μCOP; Figures 1a and S1).^44^ Typical experiments were performed in square-shaped channels with dimensions of 5.0 × 5.0 × 0.2 mm^3^ (volume = 5.0 μL; Figure S2 for the detailed geometry). Photopolymerizable resins containing acrylamide, *N*,*N*′*-*methylenebis­(acrylamide), different methacrylated single-stranded DNA, and the photoinitiator lithium phenyl-2,4,6-trimethylbenzoylphosphinate (LAP) served as hydrogel precursors, which were polymerized into hydrogel posts by patterned UV light (415 nm) projected through a digital micromirror device. Methacrylated DNA copolymerizes during this process and becomes covalently immobilized within the hydrogel network. Subsequent resin exchange allows the introduction of a different DNA-functionalized hydrogel post at different positions (Figure S3). The confidence of the printing process can be visualized by immobilizing dye-labeled complementary DNA to the posts and subsequent fluorescence microscopy (Figure 1b). The fabricated arrays consist of uniform cylindrical posts with diameters of ∼200 μm and heights of ∼100 μm (Figure 1c,d). The lower height of the posts compared to the channel height (200 μm) is attributed to the oxygen-permeability of the PDMS that is used as top layer, which inhibits polymerization. The amount of DNA in a single post polymerized from a pregel solution with a DNA concentration of 50 μM can be roughly estimated as 0.16 pmol, assuming that the pregel solution is fully converted into hydrogels without volume changes.

**Figure 1:**
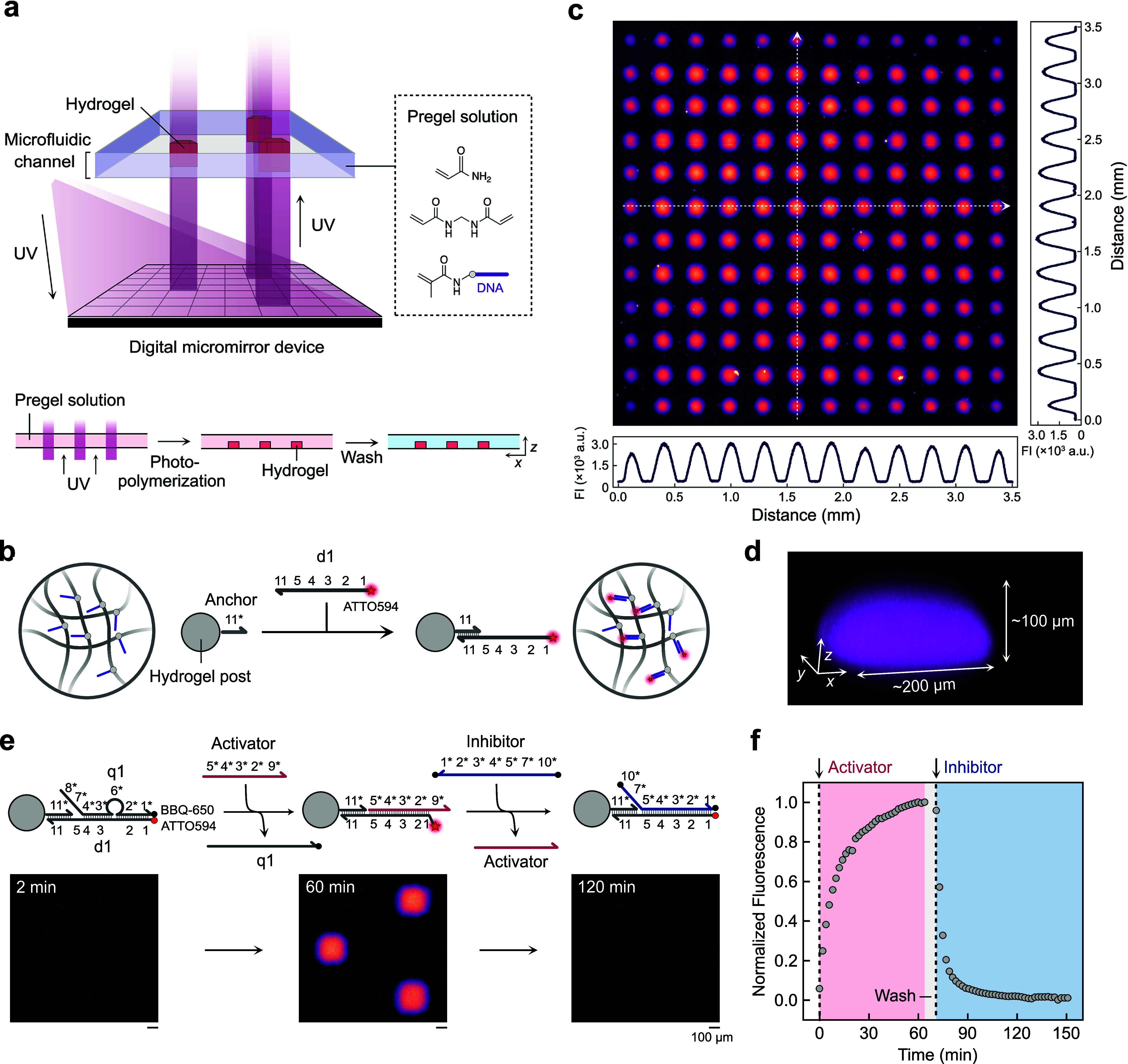
Micropatterned hydrogel post arrays serving as platforms for DNA-based SDRs. (a) Fabrication of hydrogel post arrays inside a microfluidic channel via μCOP. (b) Hybridization of ATTO594-labeled DNA strands with anchor DNA covalently attached to the hydrogel networks. (c) Widefield fluorescence microscopy images of hydrogel post arrays with cross-sectional fluorescence profiles along the *x* and *y* axes. White dotted arrows denote the positions of the cross sections used for the intensity measurements. Fluorescence originates from ATTO594-labeled DNA hybridized with anchor strands. (d) 3D-reconstructed image of hydrogel posts based on confocal laser scanning microscopy. (e) Reaction scheme and widefield fluorescence microscopy images showing the activation and inhibition of printed posts triggered by externally added DNA through SDRs. (f) Time-course of normalized fluorescence intensity averaged over three hydrogel posts, normalized between 0 and 1 based on the minimum and maximum fluorescence values.

All reaction networks used in this study rely on DNA strand displacement reactions (SDRs).^45,46^ As a first step, we confirmed that the printed hydrogel posts serve as reaction sites for such SDRs (Figure 1e,f). To this end, we printed three hydrogel posts in a triangular arrangement, with each post spaced 900 μm apart. After printing, we functionalized them with prehybridized d1/q1 double-stranded DNA by incubation with a 17 μM solution for 1 h inside the channel. The channel was then washed and kept in TAE buffer supplemented with 12.5 mM magnesium acetate. The d1 strand carries an ATTO594 fluorophore at its 5′ end, which is initially quenched by a BBQ-650 quencher attached to the 3′ end of the q1 strand. By manually introducing a complementary strand, termed Activator, the Activator displaces the q1 strands on d1 via toehold-mediated SDR between domain 5 on d1 and its complementary 5* region on the Activator. As the posts release the quencher-modified q1, their fluorescence rapidly increases within ∼60 min. The functionalized posts can participate in subsequent reactions. To illustrate this, after washing away the displaced q1, a second strand, termed Inhibitor, was subsequently added. The Inhibitor hybridizes with the newly exposed toehold region (domain 1) on d1, removing the prehybridized Activator. Since the Inhibitor also carries a BBQ-650 quencher at its 3′ end, the fluorescence of the posts is rapidly quenched again. The inhibition proceeds faster than the activation, likely due to better accessibility of the toehold region, which is of similar length in both SDRs. These results demonstrate the fundamental working principle of DNA SDRs within our micropatterned hydrogels and provide access to further DNA-mediated functions embedded within the posts.

### Transient Pattern Formation

We first demonstrate how a uniform array of DNA-functionalized hydrogel posts can generate transient fluorescence patterns in response to spatially controlled addition of activation and inhibition signals (Figure 2). For this purpose, we fabricated an array consisting of 12 × 12 identical hydrogel posts, each carrying the same DNA, in a square-shaped microfluidic channel (Figure 2a). The channel has two circular inlets, which we use to separately add two functional DNA solutions as inputs, an Activator and an Inhibitor. Once small volumes of concentrated inputs (1.0 μL, 56 μM) are introduced without applying pressure to the prefilled buffer, the DNAs slowly diffuse into the channel and trigger reactions on the hydrogel post array. Regions close to the Activator inlet exhibit strong activation, while those near the Inhibitor inlet undergo suppression. Transient fluorescence patterns emerge as the two gradients overlap and interact over time. Even though DNA diffuses more slowly inside the hydrogel posts than in the surrounding solution (diffusion coefficients *D*_gel_/*D*_solution_∼0.32; see Supporting Methods), diffusion inside the posts is still sufficiently fast, and reactions within the gel proceed rapidly, as the posts with shorter heights arranged with sufficient spacing provide large surface areas in contact with the surrounding solution without obstructing diffusion in the channel. As a result, the macroscopic activation patterns of the post array closely follow the diffusion of Activator and Inhibitor in the surrounding solution.

**Figure 2:**
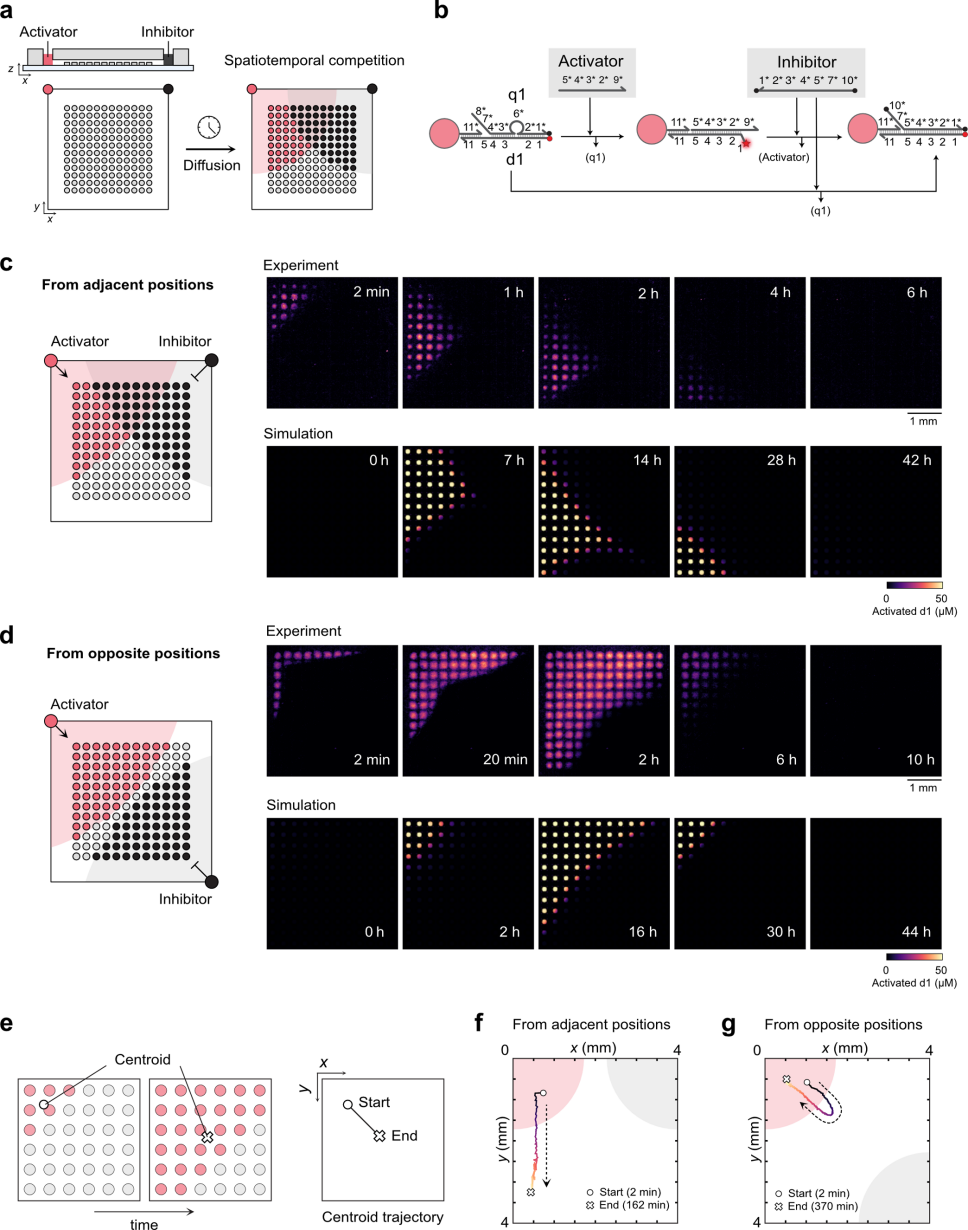
Diffusion of locally added DNA signals generates distinct transient activation patterns. (a) Conceptual diagram of local activation and inhibition in a hydrogel post array. Locally added Activator and Inhibitor strands diffuse into the microfluidic channel and react with the posts, resulting in transient activation patterns. (b) Reaction mechanism of activation and inhibition on printed posts. Posts are activated by an Activator and subsequently quenched by an Inhibitor. (c, d) Widefield fluorescence microscopy images (top) and reaction-diffusion simulations (bottom) showing transient activation patterns when two input solutions are added from (c) adjacent or (d) opposite corners. See Supporting Video 1. (e) Workflow of centroid trajectory determination from time-lapse images. An array of 6 × 6 posts is illustrated for simplicity. See Supporting Methods for details. (f, g) Centroid trajectories obtained from time-lapse fluorescence images of transient activation patterns when two input solutions are added from (f) adjacent or (g) opposite corners.

Figure 2b illustrates the reaction scheme for the activation and inhibition mechanism. As in Figure 1e, the Activator increases the fluorescence intensity of the posts, which is quenched by the Inhibitor. The Inhibitor can also hybridize with the initial d1/q1 state via the toehold domain (domain 5) on d1 and inactivates the posts but does not change fluorescence intensity.

We examined two configurations of transient activation: input solutions added from adjacent inlets and from opposite inlets. Figure 2c shows the case where the Activator is added from the top left and the Inhibitor from the top right corner (Supporting Video 1). As shown in the time-lapse images, activation begins near the Activator inlet and expands toward the bottom right as the Activator diffuses. Meanwhile, the Inhibitor diffuses from the top right, gradually erasing the activated region. Inhibition eventually becomes dominant, and the pattern disappears completely about 6 h after DNA addition. When the Activator is added from the top left and the Inhibitor from the bottom right corner (Figure 2d and Supporting Video 1), activation first expands in a similar manner and reaches its maximum size around 2 h. The pattern is then quenched from the bottom right side and vanishes after approximately 10 h. These results demonstrate that the hydrogel posts sense and discriminate the location and sequence of locally added signals, converting them into distinct transient fluorescence patterns.

We performed reaction-diffusion simulations by solving the corresponding reaction-diffusion equations in two dimensions (Figure 2c,d; see Figure S4 and Supporting Methods). The simulated patterns reproduce the observed spatiotemporal evolution—activation, directional shift, and eventual disappearance—consistent with the experimental results. The experimentally observed kinetics are faster than those predicted by the simulations, probably because the 2D model does not incorporate the enhanced reactivity of the hydrogel posts due to their exposed upper surfaces in contact with the surrounding solution.

To characterize the time development of fluorescence patterns, we calculated the centroid of the overall fluorescence pattern in each time-lapse image. Repeating this process for all frames yields a trajectory of the centroid over time, akin to a visual fingerprint of the kinetics and spatial input scenario (Figure 2e; see Supporting Methods). When the two inputs diffuse from adjacent positions (Figure 2f), the resulting trajectory becomes a straight line from top to bottom, consistent with the vector sum of the two diffusion directions. In contrast, when the inputs are added from opposite positions (Figure 2g), the trajectory first moves from the top left toward the center of the channel and then returns toward the top left.

Overall, these results confirm that locally added molecular signals can be sensed and distinguished by the hydrogel post array, which converts them into distinct, transient spatiotemporal fluorescence patterns and distinct centroid fingerprint trajectories that allow for discerning spatial signaling landscapes.

### Spatially Biased Activation by Localized Catalysts

Next, we demonstrate that printed posts can also trigger local activation through communication mediated by catalytic reaction networks (Figure 3). To this end, we implemented an entropy-driven catalytic SDR circuit adapted from earlier works^47,48^ by immobilizing selected components within hydrogel posts. As shown in Figure 3a, Catalyst posts are printed on the left side of a horizontally long microfluidic channel (10 × 2.5 × 0.2 mm^3^), while Reporter posts are printed in the rest of the channel by sequentially exposing pregel solutions to patterned UV light (Figure S3). When a mixture of Substrate and Fuel is added homogeneously to the entire channel, the Catalyst posts convert the Substrate into an Output strand at the expense of Fuel. This leads to continuous signal (Output) generation from these sender posts. The generated Output diffuses along the channel and activates the Reporter posts. Since the Catalyst posts are immobilized only on the left side, the Output is produced locally, thus creating a steep concentration gradient that leads to spatially biased activation of the Reporter posts. Figure 3b shows the reaction scheme of the entropy-driven circuit. In the presence of Catalyst, Catalyst and Fuel sequentially bind to Substrate, releasing the Output strands. The released Output then hybridizes with the initially quenched Reporter duplex, leading to fluorescence activation (see Figure S5 for the kinetic profiles in bulk solution). Since the Catalyst strand is displaced from the Substrate by the Fuel, the Catalyst is regenerated and able to catalyze multiple cycles.

**Figure 3:**
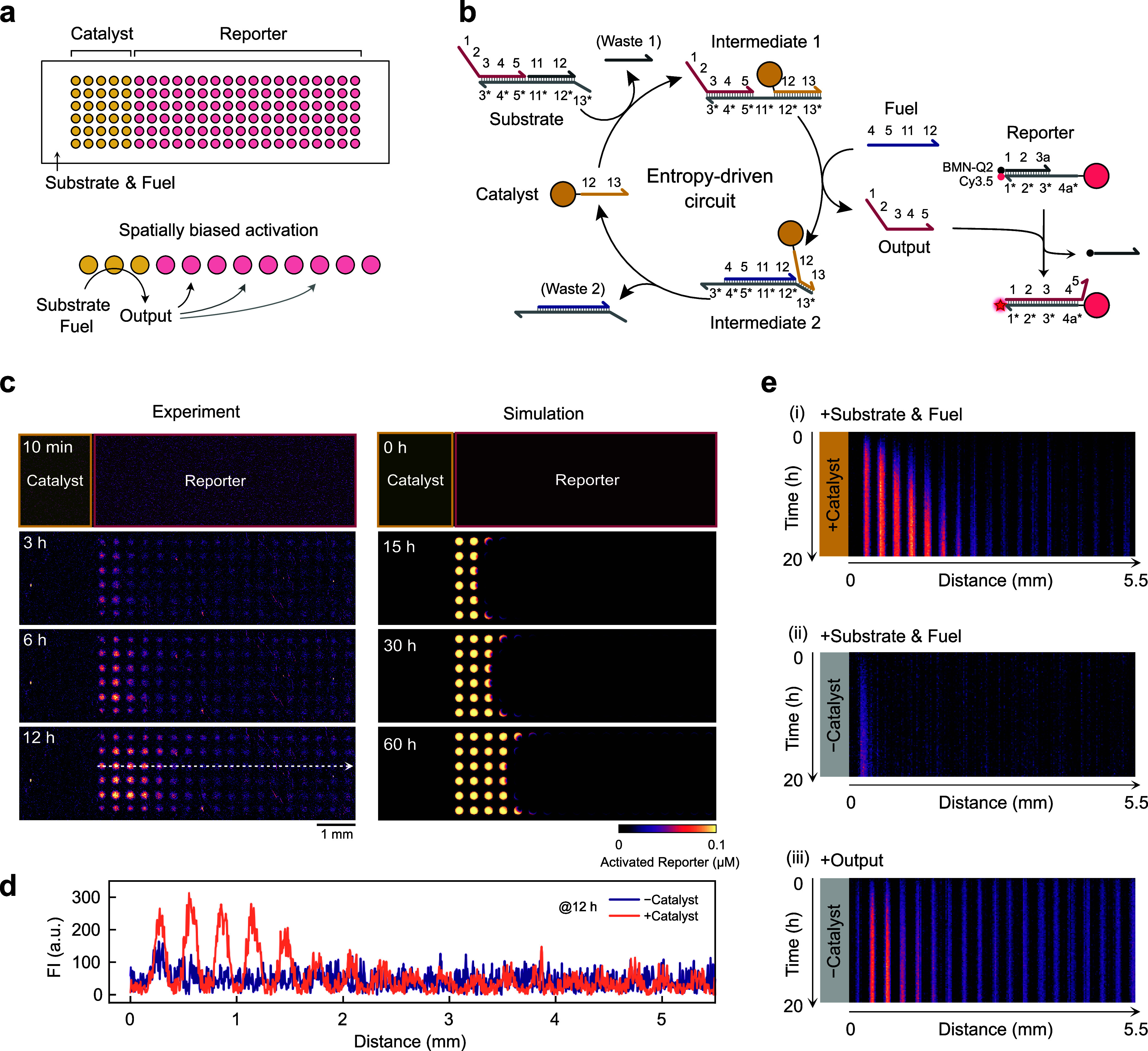
Spatially biased activation achieved by localized Catalyst posts. (a) Conceptual diagram of the catalytic activation process. Catalyst posts convert Substrate and Fuel into Output, which diffuses and activates Reporter posts in a channel. (b) Reaction scheme of the entropy-driven circuit. (c) Time-lapse widefield fluorescence microscopy images (left) and corresponding reaction-diffusion simulations (right) of the spatially biased catalytic activation. The white dotted arrow in the microscopy images indicates the cross-section lines used to generate the corresponding kymograph in (e). See Supporting Video 2. (d) Cross-sectional fluorescence intensities of Reporter posts with and without Catalyst posts, after 12 h of Substrate and Fuel additions. (e) Kymographs of cross-sectional Reporter intensities over time. Spatially biased activation is observed only when Catalyst, Substrate, and Fuel are present. Time-lapse fluorescence images of the two control experiments used to generate corresponding kymographs are summarized in Figure S7.

Figure 3c shows the time-lapse fluorescence images and the corresponding reaction-diffusion simulations (Supporting Video 2). Reporter posts are initially quenched and do not show any fluorescence. Catalyst posts are not dye-labeled, thus they do not show any fluorescence throughout the process. Reactions are initiated by introducing 0.1 μM Substrate and 0.2 μM Fuel into the channel. Once added, the Reporter array is gradually activated from the vicinity of the Catalyst array, indicating local Output generation followed by diffusion-limited activation. The corresponding reaction-diffusion simulation reproduces similar activation patterns (Figure 3c). The activation front exhibits a curved profile, advancing more rapidly at the top and bottom. This occurs because the diffusion of Output is neither physically hindered nor chemically trapped by Reporters, allowing it to propagate faster than within the array. The simulated fronts appear sharper than those observed experimentally, which can be rendered more realistic by reducing the binding rate of Output to the Reporter strand (Figure S6), while additional experimental factors may also contribute to front broadening. Cross-sectional fluorescence profiles of the Reporter array show a clearly biased activation from the left side 12 h after the Substrate and Fuel addition, while in the absence of the Catalyst, only minor leak activation is observed (Figure 3d).

To further characterize this biased activation, we generated kymographs of cross-sectional Reporter intensities over time (Figures 3e and S7). The Reporter array exhibits a spatially biased activation pattern in the presence of the Catalyst (Figure 3e (i)), while almost no activation occurs in the absence of the Catalyst (Figure 3e (ii)). When the Catalyst, Substrate, and Fuel are absent and the Output is directly added, the entire array is activated almost simultaneously, with a slight increase on the left (Figure 3e (iii)). This slight activation on the left occurs because the Reporter posts act as local sinks that deplete the Output within the array, whereas the Output remains more abundant in the Reporter-free region on the left, from which it subsequently diffuses to the right and activates nearby Reporters (see Figure S8).

Overall, these results demonstrate that immobilized Catalyst posts can locally activate Reporter posts through catalytic signal amplification, creating spatially biased activation patterns that are determined by the Catalyst location.

### Feedback-Mediated Communication

Next, we explore more complex communication behavior by implementing a negative feedback loop between two types of posts (Figure 4). This feedback process involves three consecutive steps between Posts 1 and 2 (Figure 4a): activation of Posts 1 by added Input, activation of Posts 2 by Posts 1, and inhibition of Posts 1 by Posts 2. These hydrogel posts are patterned in a 12 × 12 array in a square-shaped microfluidic channel with equal numbers of Posts 1 and 2.

**Figure 4:**
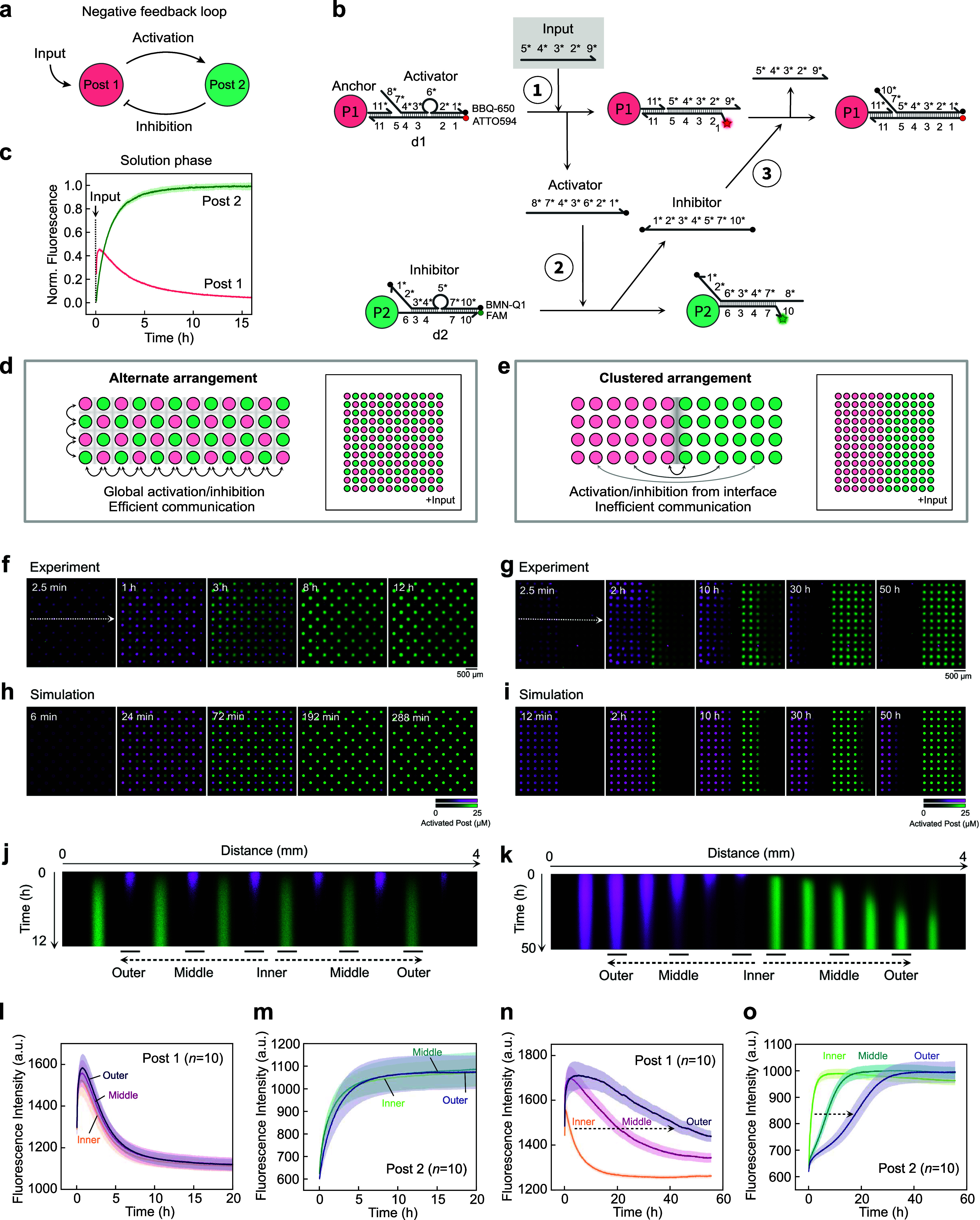
Negative-feedback-mediated communication. (a) Conceptual diagram of negative-feedback-mediated communication. (b) Reaction scheme showing bidirectional signal exchange between two types of posts initiated by input addition. (c) Fluorescence intensity changes in the bulk solution. Intensities are normalized to the theoretical maximum and minimum values determined from separate measurements of fully activated states (saturated intensities of Anchor/d1/Activator + Input for Post 1 and d2/Inhibitor + Activator for Post 2) and quenched states (Anchor/d1/Activator for Post 1 and d2/Inhibitor for Post 2), respectively. Averages and standard deviations were calculated from *n* = 6 replicates. (d, e) Schematic illustrations of communication between hydrogel posts in (d) alternate and (e) clustered arrangements. (f, g) Time-lapse widefield fluorescence images showing negative feedback-mediated communication in (f) alternate and (g) clustered arrangements. See Supporting Video 3. (h, i) Reaction-diffusion simulations of communication in (h) alternate and (i) clustered arrangements. (j, k) Kymographs generated from time-lapse cross-sectional fluorescence intensities of posts in (j) alternate and (k) clustered arrangements. (l–o) Mean time-dependent fluorescence intensities of inner, middle, and outer posts: (l, n) for Posts 1 and (m, o) for Posts 2, in (l, m) alternate and (n, o) clustered arrangements. The average intensities and standard deviations were calculated from 10 posts in the corresponding positions, excluding the uppermost and the lowermost rows, whose behaviors are significantly influenced by the surrounding solution.

Figure 4b shows the reaction scheme of the implemented negative feedback adapted from previous studies.^22,49^ Upon Input addition, the Input activates Posts 1 by displacing quencher-modified Activator strand via a toehold domain (domain 5) of d1 strand. The released Activator diffuses and reacts with Posts 2, displacing the Inhibitor strand via a toehold domain 6 and activating fluorescence. Subsequently, the released Inhibitor diffuses back to Posts 1, replacing the Input and suppressing their fluorescence again. As validated in bulk solution (Figure 4c), the DNA complex corresponding to Posts 1 (initially the Anchor/d1/Activator complex) shows a rapid increase of fluorescence intensity in 20 min followed by gradual decrease, while the DNA complex corresponding to Posts 2 (initially the d2/Inhibitor complex) displays delayed activation, reaching a plateau after approximately 12 h.

We patterned two spatial post configurations—alternate and clustered—and investigated how these arrangements affect the collective communication behavior. In the alternate arrangement (Figure 4d), each post is surrounded by posts of the other type, promoting frequent exchange of signals between Posts 1 and 2 and thus enabling efficient communication. In contrast, in the clustered arrangement (Figure 4e), effective signal exchange occurs only at the interface between the two clusters. As the distance from the interface increases, signal transmission becomes less efficient.

Figure 4f shows time-lapse fluorescence images of communication in the alternate arrangement (Supporting Video 3). Upon the Input addition, Posts 1 rapidly increase in fluorescence intensity simultaneously, reaching a maximum within 1 h after Input addition (5.6 μM). Concurrently, Posts 2 are gradually activated through the diffusing Activator and reach a maximum 8 h after Input addition. Posts 1 are subsequently quenched by the diffusing Inhibitor from Posts 2, consistent with the transient dynamics observed in solution (Figure 4c). In the clustered arrangement, where Posts 1 are located on the left half of the array and Posts 2 on the right (Figure 4g), Posts 1 are first globally activated, and then Posts 2 are gradually activated from the interface between Posts 1 (Supporting Video 3). Subsequently, Posts 1 show gradual suppression initiated from the interface. Posts 1 on the far side from Posts 2 remain partially activated even 50 h after Input addition. The corresponding reaction-diffusion simulations match the collective behavior in both alternate and clustered arrangements (Figure 4h,i).

The transient characteristics of the system are confirmed by the corresponding kymographs. In the alternate arrangement, Posts 1 and 2 show simultaneous activation and inhibition behaviors with slight edge-to-edge differences caused by small variations in post size (Figure 4j), while in the clustered arrangement, Posts 1 and 2 display spatially propagating inhibition and activation patterns, respectively, originating from their interface (Figure 4k). Mean fluorescence intensities in the alternate arrangement calculated for inner, middle, and outer posts (corresponding to first, third, and fifth columns from the center, respectively) show nearly identical time courses for both Posts 1 and 2 (Figure 4l,m), confirming uniform activation and inhibition irrespective of position. In contrast, in the clustered arrangement, inner Posts 1 display sharp transient pulses, while outer ones quench more slowly (Figure 4n); conversely, Posts 2 near the interface activate earlier than those farther away (Figure 4o). These results demonstrate that the spatial arrangement of posts modulates the strength and synchronization of communication within the feedback-regulated network, allowing global or localized activation to be tuned by geometry.

The high degree of programmability in both spatial arrangement and reaction chemistry in our system enables systematic and extensive parameter studies, which we explored through reaction-diffusion simulations with experimentally accessible scales (Supporting Note 1). We examined how key parameters, including interpost distance, diffusion coefficients, and Input concentration, influence the collective dynamics. We confirm that increasing the interpost distance, decreasing diffusion coefficients, or lowering the Input concentration slows down interpost communication; however, these parameter changes do not qualitatively alter the distinct macroscopic behaviors observed in the alternate and clustered arrangements. In addition to these intuitive trends, the simulations also revealed several nontrivial collective effects arising from the interplay between geometry and reaction kinetics.

## Conclusion

In summary, we developed a microfluidic platform using micropatterned artificial cell-like compartments to study how they respond to localized or global stimuli and how intercellular communication can be programmed by their arrangements. Our hydrogel-based compartments function as scaffolds for various DNA SDRs, enabling dynamic activation patterns that follow the diffusion of locally introduced activator and inhibitor signals, enabling to dissect spatially organized signaling landscapes. By incorporating catalytic reaction networks, we generate concentration gradients, leading to efficient signal amplification and transmission to reporter cells. Additionally, by introducing a negative feedback loop, we achieve nonlinear communication dynamics among the artificial cellular units. Furthermore, we show that spatial arrangement modulates communication efficiency, allowing the systems to emerge with fundamentally different collective dynamics under otherwise identical chemistry. Taken together, these three systems allowed us to examine how the interplay between geometry and reaction kinetics shapes macroscopic spatiotemporal patterns across complex chemical reaction networks.

From a materials design perspective, this study highlights the simplicity of fabricating microfluidic platforms and the flexibility of reaction network design. All implemented reaction networks are based solely on strand displacement chemistry, ensuring robust operation under diverse conditions. Our work opens the door to exploring a wider range of spatial configurations, which may help optimize communication architectures for specific computational or sensing purposes. Furthermore, although all the presented systems are designed for single-use operation, the implementation of resetting strategies and/or continuous fuel supply and digestion mechanisms may enable multiple activation-inhibition cycles and more advanced self-oscillatory systems.

From a biophysical and synthetic biology perspective, our platform provides a simple experimental model for studying how the spatial organization of cells influences collective behaviors in chemical and biological networks. The geometry-governed dynamics emerge across three chemically distinct network motifs, indicating that spatial positioning acts as an independent design parameter, rather than a system-specific effect. The local signaling, catalytic reactions, and negative feedback implemented here are universal mechanisms also found in living systems, and this minimal yet tunable platform enables to experimentally explore how the arrangement of such reaction units influences macroscopic communication behavior.

## Materials and Methods

### Materials

All oligonucleotides except methacrylated Anchor (MAcryl-Anchor) strand were purchased from Biomers (see Table S1 for the list of DNA sequences). 1*H*,1*H*,2*H*,2*H*-perfluorooctyltriethoxysilane, acrylamide (AAm, ≥99%) and *N*,*N*′-methylenebis­(acrylamide) (Bis, ≥99%) were purchased from Fischer Scientific. 50 × TAE buffer was purchased from Thermo Scientific. Magnesium acetate tetrahydrate (≥99%) was purchased from Carl Roth. The purchased chemicals were used without further purification. Lithium phenyl-2,4,6-trimethylbenzoylphosphinate (LAP) and MAcryl-Anchor DNA were synthesized based on published literature.

### Synthesis Procedures

#### Synthesis of LAP

The synthesis was carried out according to a previously reported procedure.^50^ Briefly, ethyl (2,4,6-trimethylbenzoyl) phenylphosphinate (5.69 g, 18.0 mmol) was dissolved in 2-butanone (100 mL). Lithium bromide (6.25 g, 72.0 mmol) was added, and the mixture was heated to 50 °C for 10 min, allowed to cool, and left to stand overnight. The crystallized product was recovered by filtration, washed with ice-cold 2-butanone, and dried in vacuo to yield LAP as a fine white powder in quantitative yield.

#### Synthesis of MAcryl-Anchor DNA

The solid-phase oligonucleotide synthesis was carried out according to the previously reported protocol^36^ using a K&A Laborgeräte H-8 synthesizer. The synthesized oligonucleotide was purified by High Performance Liquid Chromatography (HPLC) on a Dionex Ultimate 3000 (Thermo Fischer Scientific).

### Fabrication of Microfluidic Channels

Microfluidic channels with dimensions of 5.0 × 5.0 × 0.2 mm^3^ were used in most experiments, except for those in Figure 3, where 10 × 2.5 × 0.2 mm^3^ microfluidic channels were used. These channels were composed of polydimethylsiloxane (PDMS) layers sculpted from positive molds and sealed with glass slides on the bottom surface.

The molds were designed in Blender (version 3.5.0) and OpenSCAD (version 2021.01) and fabricated using an Asiga MAX X27 digital light processing 3D printer with Moiin Tech Clear photocurable resin. The 3D-printed molds were rinsed with isopropanol, dried with nitrogen, and subsequently exposed to UV light using a Nailstar UV lamp (four 9 W bulbs, peak wavelength λ = 365 nm). The molds were then placed in an oven at 150 °C for 2 h. After heating, the molds were plasma-treated for 30 s and then fluorinated by placing them with 1*H*,1*H*,2*H*,2*H*-perfluorooctyltriethoxysilane in a desiccator for 30 min to prevent PDMS replicas from binding to the molds.

To fabricate microfluidic chips, a mixture of PDMS monomer and curing agent (Sylgard 184, silicone elastomer kit, Dow Corning) at a 10:1 weight ratio was poured on the printed molds and degassed under vacuum for 1 h. The molds were then cured on a hot plate set at 100 °C for 2 h. After cooling, the PDMS replicas were carefully peeled off from the molds. Inlet and outlet holes were created at the corners of PDMS channels using a biopsy punch with a diameter of 2.0 mm for the experiments in Figure 2 and 1.2 mm for the rest of the experiments. Immediately before use, microfluidic chips were assembled by plasma-treating both the PDMS and a VWR microscope glass slide (76 × 26 × 1 mm^3^) and bringing the treated surfaces into contact. The assembled chips were heated on a hot plate at 100 °C for 2 min to ensure strong chemical bonding.

### Fabrication of Hydrogel Post Arrays in Microfluidic Channels

Hydrogels were prepared by UV-initiated free radical copolymerization of AAm, Bis, and MAcryl-DNA. Stock solutions of AAm, Bis, photoinitiator LAP, and DNA were prepared in TAE buffer (40 mM tris-acetate, 1 mM EDTA) supplemented with 12.5 mM magnesium acetate tetrahydrate (TAE/Mg^2+^). Unless otherwise noted, the TAE/Mg^2+^ buffer was used as the common buffer for all reactions. The pregel solution contained 1.41 M AAm, 10 mM Bis, 1 wt % LAP, and MAcryl-DNA, whose sequence and concentration differed depending on experiments. The pregel solution was introduced into a microfluidic channel, and patterning was performed by projecting a UV pattern onto the channel. For projection, we used a Wintech PRO4500 Optical Engine (415 nm, 25 mW/cm^2^), with 58 × 58 μm^2^ pixels and a 65.6 × 41.0 μm^2^ field of view. After gel formation, the unpolymerized resin was removed by flushing buffer through the channel. For platforms with two types of hydrogels, a second pregel solution was injected for an additional printing cycle.

### Visualization of Hydrogel Post Array

A 12 × 12 array of hydrogel posts with 310 μm spacing was printed in a microfluidic channel. The pregel solution contained 50 μM MAcryl-Anchor. After printing and washing out the unpolymerized resin, the channel was filled with 14 μM of ATTO594-labeled d1 solution and incubated for 1 h to visualize hydrogel posts by forming Anchor/d1 duplexes within the posts, followed by washing with buffer.

### Activation and Inhibition by Externally Added DNA Signals

A 12 × 12 array of hydrogel posts with 310 μm spacing was printed in a microfluidic channel. The pregel solution contained 50 μM MAcryl-Anchor. After printing and washing out the unpolymerized resin, the channel was filled with 17 μM of premixed d1/Activator solution (mixed 1:1) and incubated at room temperature for 1 h to allow the formation of Anchor/d1/q1 complexes within hydrogel posts, followed by washing with buffer. Subsequently, the channel was filled with 1.1 μM Activator solution to activate the posts. The reaction was terminated by washing the channel with buffer 64 min after the Input addition. Then, 1.1 μM Inhibitor solution was introduced into the channel to initiate the deactivation of posts.

### Transient Pattern Formation

A 12 × 12 array of hydrogel posts with 310 μm spacing was printed in a microfluidic channel. The pregel solution contained 50 μM MAcryl-Anchor. As in the homogeneous activation and inhibition experiments shown in Figure 1e, the channel was filled with 17 μM of premixed d1/Activator solution (mixed 1:1) and incubated at room temperature for 1 h to allow the formation of Anchor/d1/q1 complexes within hydrogel posts, followed by washing with buffer. After functionalization, 1.0 μL of 56 μM Activator solution was gently added to one inlet, and an equal amount of Inhibitor solution was added to the other inlet without applying pressure to the prefilled buffer in a channel. The Inhibitor was always added first, followed by the Input within 30 s. The channel was then sealed with a cover glass, and the time-lapse fluorescence was monitored.

### Spatially Biased Activation by Catalysts

Hydrogel posts were printed in a microfluidic channel by sequentially exposing pregel solutions to patterned UV light. First, a 6 × 23 post array was printed from the Reporter pregel solution. Next, a 6 × 5 post array was printed from the Catalyst pregel solution, aligned with the previous one to form a 6 × 28 array with 310 μm spacing. The pregel solution for the Catalyst posts contained 2.0 μM MAcryl-Catalyst, whereas that for the Reporter posts contained 2.0 μM MAcryl-Reporter. In the control experiment without Catalyst, no MAcryl-Catalyst was mixed in the Catalyst pregel solution. After printing, the channel was filled with 94 μM of BMN-Q2-labeled complementary strand to quench the Reporter posts and washed after 1 h of incubation at room temperature. The reaction was initiated by homogeneously adding a mixture of 0.1 μM Substrate and 0.2 μM Fuel. The Substrate complex was annealed beforehand by heating up to 95 °C and cooling to 20 °C at a rate of 1 °C/min using an Eppendorf ThermoMixer C. For the positive control experiment, 0.1 μM Output strand was added instead. The channel was then sealed with a cover glass, and the time-lapse fluorescence was monitored.

### Negative-Feedback-Mediated Communication

Hydrogel posts were printed in a microfluidic channel by sequentially exposing pregel solutions to patterned UV light. In a 12 × 12 array, equal numbers of Post 1 and Post 2 were patterned with 310 μm spacing in two distinct arrangements: alternate and clustered (Figure 4d,g). Posts 2 were printed first (72 posts), followed by Posts 1 (72 posts). The pregel solution for Posts 1 contained 50 μM MAcryl-Anchor, whereas that for Post 2 contained 50 μM MAcryl-d2. After printing, the channel was filled with 14 μM Inhibitor solution and incubated for 30 min to quench Posts 2. After washing out the unhybridized Inhibitor, the microfluidic chip was placed on ice, and the channel was filled with 4.2 μM d1/Activator duplex (premixed 1:1 in TAE buffer supplemented with 50 mM magnesium acetate) and incubated on ice for 20 min to yield Anchor/d1/Activator complex in Posts 1. The channel was then washed with TAE buffer with 50 mM magnesium acetate, followed by the common TAE/Mg^2+^ buffer. The incubation on ice in a high-salinity buffer was conducted to stabilize the prehybridized d1/Activator complex in solution and d2/inhibitor complex in Posts 2, thereby preventing unwanted hybridization to form d1/Inhibitor and d2/Activator (Figure S9). After functionalization, the channel was filled with 5.6 μM Input in TAE/Mg^2+^ buffer and sealed with a cover glass, and time-lapse fluorescence was monitored.

### Fluorescence Microscopy

All reactions were monitored at room temperature. Figure 1c,e were captured using an EVOS M7000 fluorescence microscope (Thermo Fisher Scientific) equipped with a 4× objective. ATTO594-labeled species were imaged using the Cy5 channel (excitation: 635 nm, emission: 692 nm). Images were acquired using associated software (version 2.1.677.717). 3D image of a single hydrogel post shown in Figure 1d was captured using a Leica Stellaris 5 confocal laser scanning microscope equipped with a 5×/0.15NA dry objective. ATTO594-labeled species were imaged using 561 nm excitation. Z-stack images were collected at 6.13 μm over a total height of 368 μm, resulting in 60 optical sections. Images were acquired using LAS X software (version 4.3.0.24308). Figure 2c,d were captured using a Dino-Lite AM4115T-GRFBY digital microscope. ATTO594-labeled species were imaged using a TxRed/mCherry channel (excitation: 575 nm, emission: 610 nm). Images were acquired using DinoCapture 2.0 software (version 1.5.49.B). Figures 3c and 4 f,g were captured using a Nikon SMZ25 stereo microscope. FAM- and Cy3.5-labeled species were imaged using 555 nm LED, and ATTO594-labeled species were imaged using 640 nm LED. Images were acquired using NIS-Elements AR software (version 5.21.03).

### Fluorescence Spectroscopy

Negative feedback behavior was confirmed in bulk solution (Figure 4c) using a Tecan Spark plate reader in top mode. Samples were prepared in a black 384-well plate (Costar, Corning) using a Dispendix I.DOT liquid handler. Anchor/d1/Activator and d2/Inhibitor were first prepared by mixing the respective strands at a (1:)­1:1 molar ratio and used without annealing. Each complex was then mixed to a final concentration of 47 nM and a final volume of 20 μL for each complex. The reaction was initiated by adding 94 nM Input, and fluorescence was recorded every 1 min at 20 °C. To minimize evaporation, 5 μL of hexadecane was added on top of each well. Excitation/emission wavelengths were set to 485/535 nm for FAM and 560/610 nm for ATTO594, respectively. Averages and standard deviations were calculated from *n* = 6 replicates. Reference samples for the maximum and minimum fluorescence used for normalization were measured in parallel. For Post 1, maximum and minimum values were obtained from Anchor/d1/Activator + Input and Anchor/d1/Activator alone; for Post 2, from d2/Inhibitor + Activator and d2/Inhibitor alone. All reference samples were prepared at the same final concentrations as the main reactions: 47 nM Anchor/d1/Activator or d2/Inhibitor, 94 nM Input, and 47 nM Activator (corresponding to the theoretical maximum amount released from Anchor/d1/Activator). Fixed maximum and minimum values for normalization were obtained by averaging the reference plots (*n* = 3 each) and then taking the mean of the plateau values after the signals had reached saturation (≥3 h after mixing).

### Reaction-Diffusion Simulation

COMSOL Multiphysics 6.3 was used to simulate the reaction-diffusion dynamics in two dimensions. Transport of Diluted Species and Chemistry modules were used as our base models. Details are described in Supporting Methods and Table S2.

### Image Processing and Analysis

All image processing was performed using Fiji (version 2.16.0). For the time-lapse images shown in Figure 2, background inhomogeneity was corrected using the BaSiC plugin^51^ based on a flat-field correction algorithm. The final frames after the complete disappearance of the patterns were used as blank images, which were subtracted from all other time points to remove static background fluorescence. For the images shown in Figure 3, background fluorescence was removed by subtracting the initial frame from all other time points. The last 5 columns of the Reporter posts were cut off from the right side for better visibility of images. For the images shown in Figure 4, background fluorescence was removed by subtracting blank images acquired before the Input addition.

Fluorescence intensities of hydrogel posts were quantified using Fiji. The mean pixel intensity within each post was calculated by manually selecting the corresponding regions. Cross-sectional intensity profiles were obtained by calculating average pixel intensities along lines with a width of 5 pixels, from which kymographs were generated using a custom Python script (available upon request). Centroid trajectories were obtained by repeatedly calculating the centroid positions from binarized time-series images using a custom Python script (see Supporting Methods for details).

## Supplementary Material









## Notes

Details of theoretical calculations, reaction-diffusion simulations, and image analysis; fluorescence spectroscopy and microscopy data of control experiments; geometries of microfluidic channels; lists of DNA sequences; parameter studies of negative-feedback-mediated communication (PDF)
Localized signals create distinct activation patterns (Video S1) (MP4)
Localized catalysts create spatially biased activation patterns (Video S2) (MP4)
Arrangement determines communication efficiency (Video S3) (MP4)

## Abbreviations

μCOP: microscale continuous optical printing
AAm: acrylamide
Bis: *N*,*N*′-methylenebis­(acrylamide)
LAP: lithium phenyl-2,4,6-trimethylbenzoylphosphinate
PDMS: polydimethylsiloxane
SDR: strand displacement reaction.
