# Supplementary material for: Micropatterned DNA Hydrogels for Spatiotemporal Programming of Chemical Reaction Networks
Source: ACS Nano. 2026 Mar 14;20(12):9913–24. doi: 10.1021/acsnano.5c20505 (PMC13045365; doi:10.1021/acsnano.5c20505)
Supplement: Supplementary file 1 [file nn5c20505_si_001.pdf]

Supporting Information for:

Micropatterned DNA Hydrogels for Spatiotemporal Programming of Chemical Reaction Networks

Kohei Nishiyama, Piet J. M. Swinkels, Brigitta Dúzs, Weixiang Chen, Andreas Walther*

Life-Like Materials and Systems, Department of Chemistry, University of Mainz, Germany

*Corresponding Author: andreas.walther@uni-mainz.de

Table of Contents

1. Supporting Methods 2

1.1 A priori estimation of diffusion coefficient for ssDNA in hydrogels 2

1.2 Reaction-diffusion simulations 2

1.2.1 General setting 2

1.2.2 Transient pattern formation (relates to Figure 2) 2

1.2.3 Spatially biased catalytic activation (relates to Figure 3) 3

1.2.4 Negative-feedback-mediated communication (relates to Figure 4) 4

1.3 Generation of centroid trajectory 5

2. Supporting Figures 6

3. Supporting Tables 10

4. Supporting Note 1 12

4.1 Parameter studies of negative-feedback-mediated communication 12

4.1.1 Distance between posts 12

4.1.2 Diffusion coefficient of ssDNA in hydrogel posts 14

4.1.3 Diffusion coefficient of ssDNA in both posts and solution 16

4.1.4 Input concentration 18

5. Supporting Videos 21

References 21

1. Supporting Methods

1.1 A priori estimation of diffusion coefficient for ssDNA in hydrogels

The diffusion coefficient of ssDNA in hydrogels (D_g) can be estimated using an equation based on Brinkman's equation.[1] In the original form, the hindrance factor is described as a function of the solute radius r_s and the hydraulic permeability k. By approximating the effective solute size with the radius of gyration ($r_s \approx R_g$) and defining the effective mesh size as $\xi_H \equiv \sqrt{k}$, the equation can be expressed as:

$$\frac{D_g}{D_0} = \left[1 + \frac{R_g}{\xi_H} + \frac{1}{3}\left(\frac{R_g}{\xi_H}\right)^2\right]^{-1} \tag{1}$$

where D_0 is the diffusion coefficient of ssDNA in free solution. ξ_H of poly(acrylamide) used in this study is estimated to be ~2.1 nm using the empirical equation $\xi_H = \phi^{-0.33}$, where ϕ is the polymer volume fraction (~0.102).[2] The R_g of 46 nt ssDNA (average length of diffusible ssDNA used in this study) is known to be ~3.0 nm.[3] Substituting these values into this formula yields a relative diffusion coefficient D_g/D_0 of ~0.32.

1.2 Reaction-diffusion simulations

1.2.1 General setting

We used COMSOL Multiphysics 6.3 to simulate the reaction-diffusion dynamics in two dimensions. Transport of Diluted Species module and Chemistry module were used as our base models. No-flux boundary conditions were applied to the channel walls, whereas free diffusions were applied for the post borders. The mesh resolution was set to "Finer" in COMSOL's predefined settings. The time-dependent study was solved using COMSOL's default solver (Backward Differential Formula (BDF) method). The relative and absolute tolerances were set to 0.005 and 0.1, respectively. All kinetic rate coefficients, diffusion coefficients, and initial concentrations used in simulations are summarized in Table S2.

1.2.2 Transient pattern formation (relates to Figure 2)

The reaction-diffusion model is based on the following three chemical reactions:

$$\mathrm{P_1 + Activator} \underset{k_{\mathrm{off}}}{\overset{k_{\mathrm{1on}}}{\rightleftarrows}} \mathrm{P_2 + q1} \tag{2}$$

$$\mathrm{P_2 + Inhibitor} \underset{k_{\mathrm{off}}}{\overset{k_{\mathrm{2on}}}{\rightleftarrows}} \mathrm{P_3 + Activator} \tag{3}$$

$$\mathrm{P_1 + Inhibitor} \underset{k_{\mathrm{off}}}{\overset{k_{\mathrm{1on}}}{\rightleftarrows}} \mathrm{P_3 + q1} \tag{4}$$

where k denotes the rate coefficients, and P_n corresponds to the nth state of posts. Assuming the mass action kinetics, the net reaction rates (R_i) are defined as:

$$R_1 = k_{\mathrm{1on}}[\mathrm{P_1}][\mathrm{Activator}] - k_{\mathrm{off}}[\mathrm{P_2}][\mathrm{q1}] \tag{5}$$

$$R_2 = k_{\mathrm{2on}}[\mathrm{P_2}][\mathrm{Inhibitor}] - k_{\mathrm{off}}[\mathrm{P_3}][\mathrm{Activator}] \tag{6}$$

$$R_3 = k_{1\text{on}}[\text{P}_1][\text{Inhibitor}] - k_{\text{off}}[\text{P}_3][\text{q1}] \tag{7}$$

where [ ] denotes space- and time-dependent concentrations of the chemical species. The diffusion coefficients for Activator, Inhibitor, and q1 were set to D, while the P_n species were assumed to be immobile (diffusion coefficient = 0). The reaction-diffusion system is described by the following set of coupled partial differential equations (PDEs):

$$\partial_t[\text{Activator}] = D\nabla^2[\text{Activator}] - R_1 + R_2 \tag{8}$$

$$\partial_t[\text{Inhibitor}] = D\nabla^2[\text{Inhibitor}] + R_2 - R_3 \tag{9}$$

$$\partial_t[\text{q1}] = D\nabla^2[\text{q1}] + R_1 + R_3 \tag{10}$$

$$\partial_t[\text{P}_1] = -R_1 - R_3 \tag{11}$$

$$\partial_t[\text{P}_2] = R_1 - R_2 \tag{12}$$

$$\partial_t[\text{P}_3] = R_2 + R_3 \tag{13}$$

where ∇^2 is the 2D Laplacian operator.

1.2.3 Spatially biased catalytic activation (relates to Figure 3)

The reaction-diffusion model is based on the following five chemical reactions:

$$\text{Substrate} + \text{Catalyst} \underset{k_{\text{bi2}}}{\overset{k_{\text{bi1}}}{\rightleftarrows}} \text{Intermediate 1} + \text{Waste 1} \tag{14}$$

$$\text{Intermediate 1} + \text{Fuel} \xrightarrow{k_{\text{bi2}}} \text{Intermediate 2} + \text{Output} \tag{15}$$

$$\text{Intermediate 2} \underset{k_{\text{bi1}}}{\overset{k_{\text{uni}}}{\rightleftarrows}} \text{Catalyst} + \text{Waste 2} \tag{16}$$

$$\text{Substrate} + \text{Fuel} \xrightarrow{k_{\text{leak}}} \text{Output} + \text{Waste 1} + \text{Waste 2} \tag{17}$$

$$\text{dqReporter} + \text{Output} \xrightarrow{k_{\text{rep}}} \text{doReporter} + \text{qRep} \tag{18}$$

where dqReporter and doReporter correspond to the quenched and unquenched states of Reporter posts, respectively. qRep corresponds to the quencher-labeled strand which was initially forming the Reporter duplex. Several reverse reactions are not considered because their rate coefficients are very low and can be considered as irreversible. Assuming the mass action kinetics, the net reaction rates (R_i) are defined as:

$$R_1 = k_{\text{bi1}}[\text{Substrate}][\text{Catalyst}] - k_{\text{bi2}}[\text{Intermediate 1}][\text{Waste 1}] \tag{19}$$

$$R_2 = k_{\text{bi2}}[\text{Intermediate 1}][\text{Fuel}] \tag{20}$$

$$R_3 = k_{\text{uni}}[\text{Intermediate 2}] - k_{\text{bi1}}[\text{Catalyst}][\text{Waste 2}] \tag{21}$$

$$R_4 = k_{\text{leak}}[\text{Substrate}][\text{Fuel}] \tag{22}$$

$$R_5 = k_{\text{rep}}[\text{dqReporter}][\text{Output}] \tag{23}$$

The diffusion coefficients for Catalyst, dqReporter, and doReporter were assumed to be 0 (immobile species), while for the rest of the species, we set them to D. The system is described by the following set of coupled PDEs:

$$\partial_t[\text{Substrate}] = D\nabla^2[\text{Substrate}] - R_1 - R_4 \tag{24}$$

$$\partial_t[\text{Intermediate 1}] = D\nabla^2[\text{Intermediate 1}] + R_1 - R_2 \tag{25}$$

$$\partial_t[\text{Intermediate 2}] = D\nabla^2[\text{Intermediate 2}] + R_2 - R_3 \tag{26}$$

$$\partial_t[\text{Fuel}] = D\nabla^2[\text{Fuel}] - R_2 - R_4 \tag{27}$$

$$\partial_t[\text{Output}] = D\nabla^2[\text{Output}] + R_2 + R_4 - R_5 \tag{28}$$

$$\partial_t[\text{Waste 1}] = D\nabla^2[\text{Waste 1}] + R_1 + R_4 \tag{29}$$

$$\partial_t[\text{Waste 2}] = D\nabla^2[\text{Waste 2}] + R_3 + R_4 \tag{30}$$

$$\partial_t[\text{Catalyst}] = -R_1 + R_3 \tag{31}$$

$$\partial_t[\text{dqReporter}] = -R_5 \tag{32}$$

$$\partial_t[\text{doReporter}] = R_5 \tag{33}$$

$$\partial_t[\text{qRep}] = D\nabla^2[\text{qRep}] + R_5 \tag{34}$$

1.2.4 Negative-feedback-mediated communication (relates to Figure 4)

The reaction-diffusion model is based on the following four chemical reactions:

$$\text{P1}_1 + \text{ Input } \underset{k_{\text{off}}}{\overset{k_{1\text{on}}}{\rightleftarrows}} \text{P1}_2 + \text{Activator} \tag{35}$$

$$\text{P2}_1 + \text{Activator } \underset{k_{\text{off}}}{\overset{k_{2\text{on}}}{\rightleftarrows}} \text{P2}_2 + \text{Inhibitor} \tag{36}$$

$$\text{P1}_2 + \text{Inhibitor } \underset{k_{\text{off}}}{\overset{k_{3\text{on}}}{\rightleftarrows}} \text{P1}_3 + \text{Input} \tag{37}$$

$$\text{P1}_1 + \text{Inhibitor } \underset{k_{\text{off}}}{\overset{k_{1\text{on}}}{\rightleftarrows}} \text{P1}_3 + \text{Activator} \tag{38}$$

where Pm_n correspond to the nth state of posts m. Assuming the mass action kinetics, the net reaction rates (R_i) are defined as:

$$R_1 = k_{1\text{on}}[\text{P1}_1][\text{Input}] - k_{\text{off}}[\text{P1}_2][\text{Activator}] \tag{39}$$

$$R_2 = k_{1\text{on}}[\text{P2}_1][\text{Activator}] - k_{\text{off}}[\text{P2}_2][\text{Inhibitor}] \tag{40}$$

$$R_3 = k_{2\text{on}}[\text{P1}_2][\text{Inhibitor}] - k_{\text{off}}[\text{P1}_3][\text{Input}] \tag{41}$$

$$R_4 = k_{1\text{on}}[\text{P1}_1][\text{Inhibitor}] - k_{\text{off}}[\text{P1}_3][\text{Activator}] \tag{42}$$

The diffusion coefficients for Activator, Inhibitor, and q1 were set to D, while the Pm_n species were assumed to be immobile (diffusion coefficient = 0). The system is described by the following set of coupled PDEs:

$$\partial_t[\text{Input}] = D\nabla^2[\text{Input}] - R_1 + R_3 \tag{43}$$

$$\partial_t[\text{Activator}] = D\nabla^2[\text{Activator}] + R_1 - R_2 + R_4 \tag{44}$$

$$\partial_t[\text{Inhibitor}] = D\nabla^2[\text{Inhibitor}] + R_2 - R_3 - R_4 \tag{45}$$

$$\partial_t[\text{P1}_1] = -R_1 - R_4 \tag{46}$$

$$\partial_t[\text{P1}_2] = R_1 - R_3 \tag{47}$$

$$\partial_t[\mathrm{P1}_3] = R_3 + R_4 \tag{48}$$

$$\partial_t[\mathrm{P2}_1] = -R_2 \tag{49}$$

$$\partial_t[\mathrm{P2}_2] = R_2 \tag{50}$$

1.3 Generation of centroid trajectory

The trajectory of fluorescent signal was quantified by calculating its centroid for each image in the time-series. The analysis was performed using a custom Python script with the OpenCV library.

Each raw image was first converted to a single-channel grayscale format. The image was converted to a binary image with a fixed intensity threshold and then processed with one iteration of erode/dilate cycle to eliminate noise artifacts.

The centroid of the resulting pattern in the image was determined by calculating image moments. The (i, j)th moment of an image is defined mathematically as:

$$M_{ij} = \sum_x \sum_y x^i y^j \cdot I(x, y) \tag{51}$$

where $I(x, y)$ is the pixel intensity at the coordinate (x, y), and the summations are performed over all pixels of the image.

For a binary image, the zeroth-order moment, M_{00}, is the sum of all white pixel intensities, which is proportional to the total area of the object. The first-order moments, M_{10} and M_{01}, represent the sum of intensities weighted by the x and y coordinates, respectively.

The centroid coordinates (c_x, c_y) were calculated from these moments as follows:

$$c_x = \frac{M_{10}}{M_{00}} \tag{52}$$

$$c_y = \frac{M_{01}}{M_{00}} \tag{53}$$

This procedure was repeated for each image in the time-series to generate the full trajectory.

2. Supporting Figures

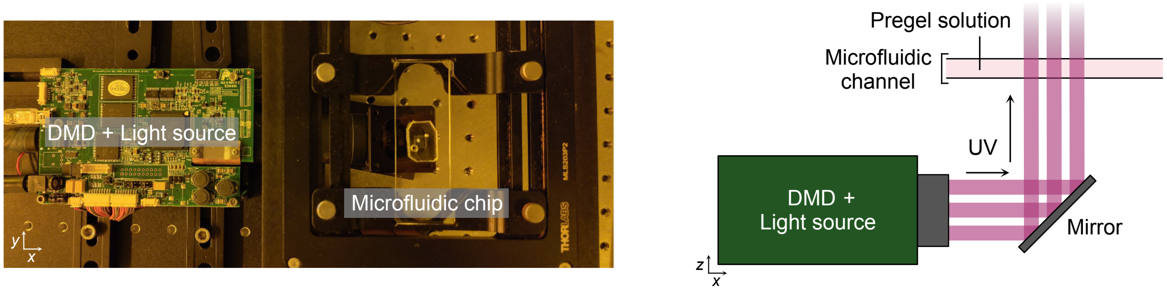

Figure S1. Digital micromirror device (DMD) setup.

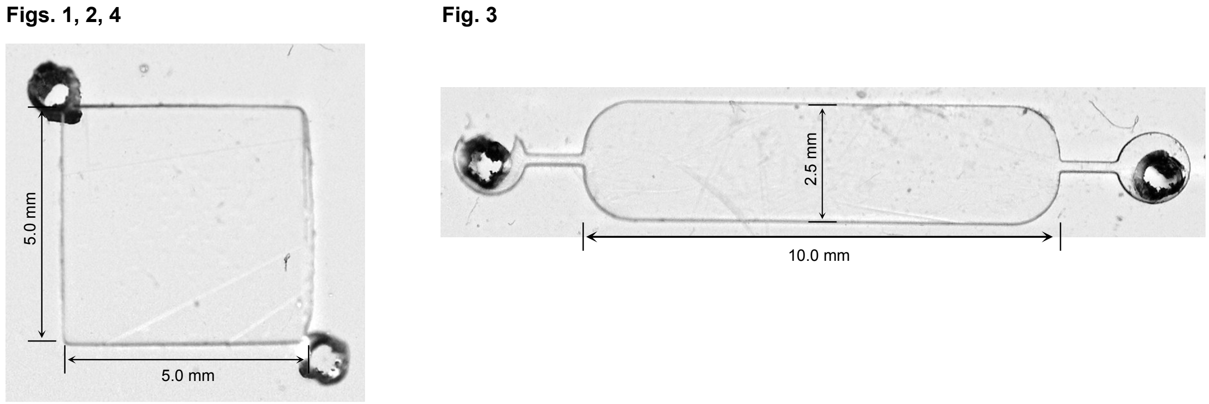

Figure S2. Geometry of microfluidic channels. Two holes were created as inlets. For the experiments shown in Figure 2, larger holes were created as reservoirs for input solutions. Both channels have heights of 0.2 mm.

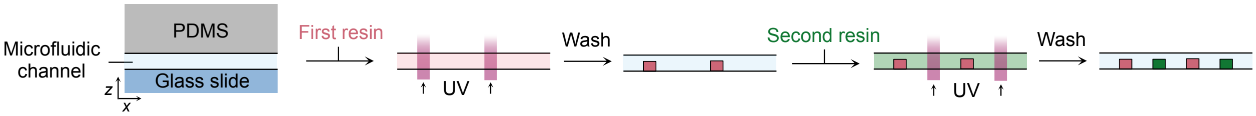

Figure S3. Multi-post printing process.

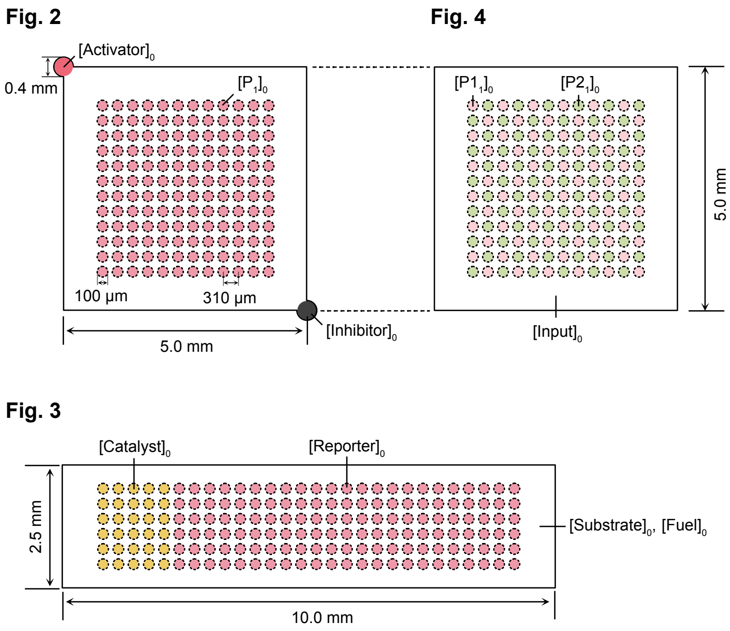

Figure S4. Geometry and initial conditions of reaction-diffusion simulations in two dimensions. No-flux boundary conditions were applied to the solid line regions, while free diffusions were applied for the dotted line regions. $[\ ]_0$ denotes the initial concentrations of chemical species.

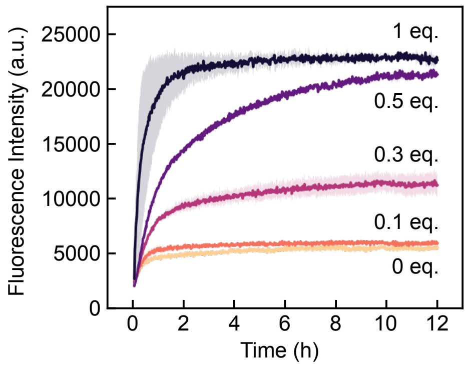

Figure S5. Catalytic signal amplification in bulk solution. Substrate and Reporter complexes were annealed in advance by mixing the respective strands at (1:)1:1 ratios by a heating-cooling cycle. 20 µL of final sample solution consists of 0.1 µM Substrate, 0.2 µM Fuel, 0.3 µM Reporter, and 0–0.1 µM Catalyst (0–1 eq.) in TAE/Mg^{2+} buffer with 5.0 µL hexadecane on top. Reactions were monitored at 20 °C. Excitation/emission wavelengths were set to 560/610 nm to record the fluorescence of Cy3.5-labeled Reporter. Averages and standard deviations were calculated from n=2 replicates. All other experimental details such as used instruments are identical to those described for Figure 4c (see Methods).

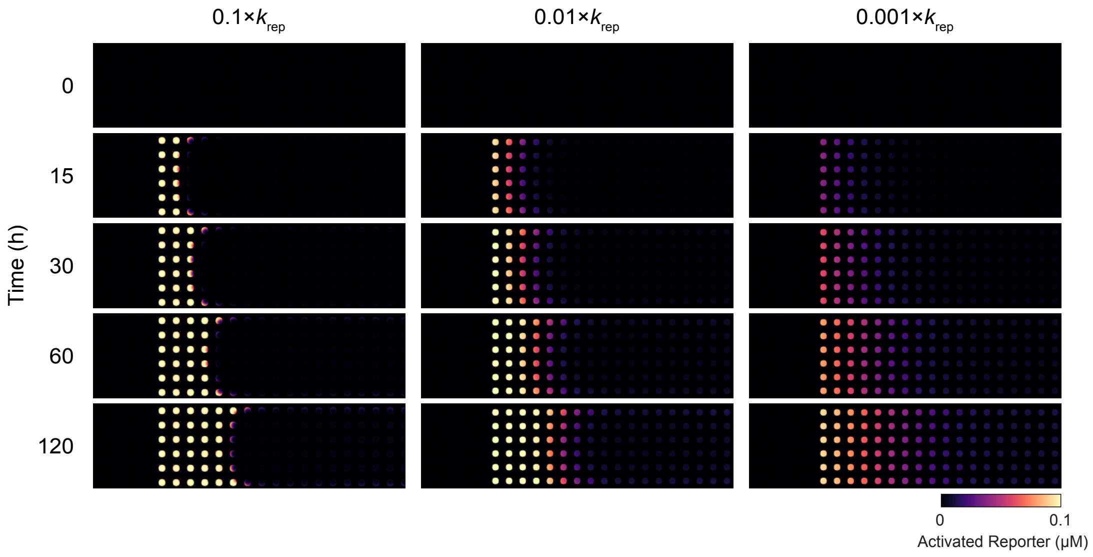

Figure S6. Effect of Output-Reporter binding rate on simulated activation fronts. Time-dependent reaction-diffusion simulations showing how the sharpness of activation fronts depends on the effect binding rate k'_{rep} of Output to the Reporter. Decreasing k'_{rep} leads to more blurred activation fronts, making the simulated profiles more consistent with experimental observations.

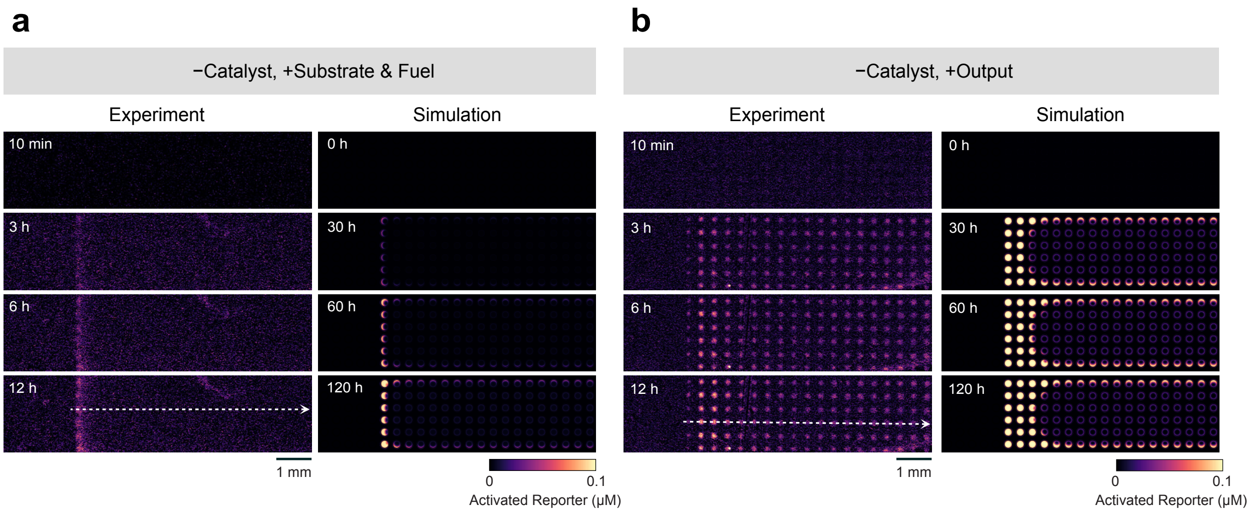

Figure S7. Control experiments for spatially biased catalytic activation. Time-lapse fluorescence images and corresponding reaction-diffusion simulations showing the activation of Reporter posts under two conditions: (a) in the absence of Catalyst posts with Substrate and Fuel present in solution, and (b) in the absence of Catalyst posts with Output present in solution. White dotted arrows in the microscopy images indicate the cross-section lines used to generate the corresponding kymographs shown in Figure 3e.

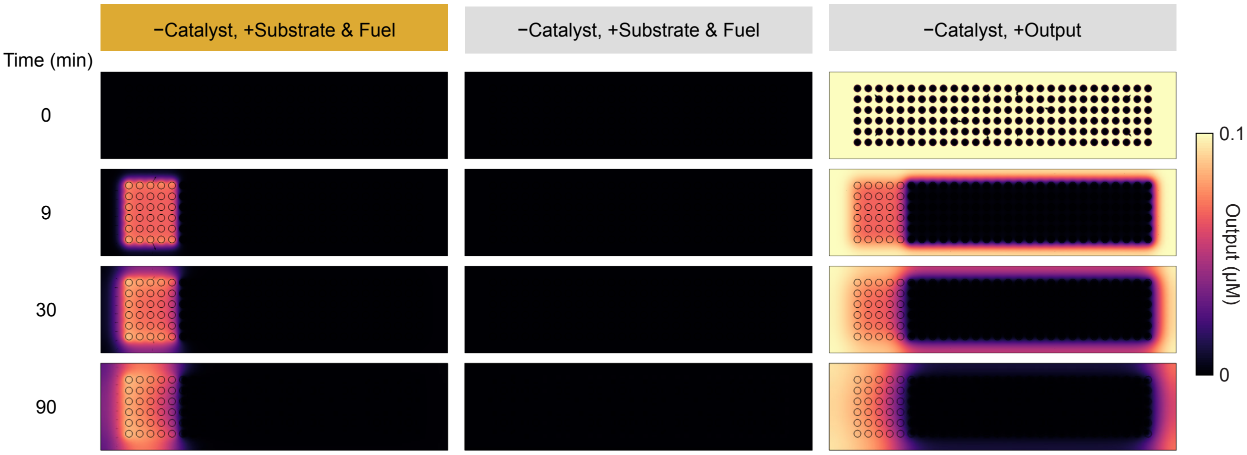

Figure S8. Time-dependent Output concentration profiles estimated from simulations. Output is locally generated only in the presence of Catalyst posts. In the absence of Catalyst, Substrate, and Fuel but in the presence of Output in solution, the Reporter posts locally deplete the Output, creating a relatively high-concentration region on the left, which results in a slightly biased activation.

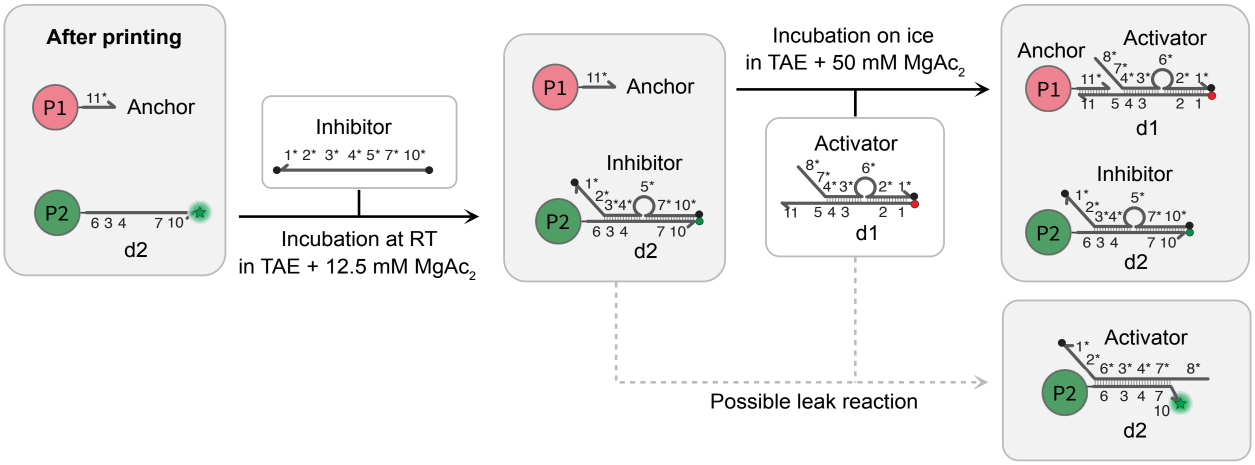

Figure S9. Functionalization process for negative-feedback-mediated communication. After functionalizing Posts 2 with Inhibitor, the d1/Activator duplex was added to form the Anchor/d1/Activator complex within Posts 1. However, under the conventional TAE/Mg^{2+} buffer (TAE buffer supplemented with 12.5 mM magnesium acetate ($MgAc_2$)) at room temperature, toehold-free strand displacement of the pre-hybridized d2/Inhibitor duplex by the Activator occurred, resulting in undesired activation of Posts 2. To prevent this, the duplex was added on ice in TAE buffer supplemented with 50 mM $MgAc_2$ which enhanced the stability of the pre-hybridized d1/Activator and d2/Inhibitor duplexes and minimized the possibility of the unwanted leak reaction.

3. Supporting Tables

Table S1. DNA sequences.

Figure	Strand	Domain (5'-3')	Sequence (5'-3')
Figs. 1-2	Anchor	11*	MAcryl/ CACAGCACGGAGGCACGACAC
	d1	1 2 3 4 5 11	GTATGTAAC GATTA GTGTAG TTAGTTAG GTGTAATATC GTGTCGTGCCTCCGTGCTGTG /Atto594
	q1	8* 7* 4* 3* 6* 2* 1*	ATTGTTCGGA TTCA CTAACTAA CTACAC ATACTTAC TAATC GTTACATAC /BBQ-650
	Activator	5* 4* 3* 2* 9*	GATATTACAC CTAACTAA CTACAC TAATC ATGGAAGATAC
	Inhibitor	10* 7* 5* 4* 3* 2* 1*	BMN-Q1/ CAACTCT TTCA GATATTACAC CTAACTAA CTACAC TAATC GTTACATAC /BBQ-650
Fig. 3	Substrate-1	13* 12* 11* 5* 4* 3*	CATGGTGA GGGTATTGTAT GAGGG TGGTTTCA GGTTGCT AGGTCTC
	Substrate-2	11 12	CCCTC ATACAATACCC
	Output	1 2 3 4 5	ATAGATCC TGATAGC GAGACCT AGCAACC TGAAACCA
	Fuel	4 5 11 12	AGCAACC TGAAACCA CCCTC ATACAATACCC
	Catalyst	0 12 13	MAcryl/ TTTTT ATACAATACCC TCACCATG
	dRep	0 4a* 3* 2* 1*	MAcryl/ TTTTT TTGCT AGGTCTC GCTATCA GGATCTAT /Cy3.5
	qRep	1 2 3a	BMN-Q2/ ATAGATCC TGATAGC GAGAC
Fig. 4	Anchor	11*	MAcryl/ CACAGCACGGAGGCACGACAC
	d1	1 2 3 4 5 11	GTATGTAAC GATTA GTGTAG TTAGTTAG GTGTAATATC GTGTCGTGCCTCCGTGCTGTG /Atto594
	d2	0 6 3 4 7 10	MAcryl/ TTTTT GTAAGTAT GTGTAG TTAGTTAG TGAA AGAGTTG /6-FAM
	Activator	8* 7* 4* 3* 6* 2* 1*	ATTGTTCGGA TTCA CTAACTAA CTACAC ATACTTAC TAATC GTTACATAC /BBQ-650
	Inhibitor	10* 7* 5* 4* 3* 2* 1*	BMN-Q1/ CAACTCT TTCA GATATTACAC CTAACTAA CTACAC TAATC GTTACATAC /BBQ-650
	Input	5* 4* 3* 2* 9*	GATATTACAC CTAACTAA CTACAC TAATC ATGGAAGATAC

- All DNA was HPLC-purified.
- MAcryl: Methacrylate modification.
- Domain 0 corresponds to T5 spacer domain to ensure sufficient distance from the methacrylate modification, which is not shown in the main figures for simplicity.

Table S2. Parameters used in reaction-diffusion simulations.

Figure	Parameter	Value	Description
General	D	3.6×10^{-7} (cm^{2}s^{-1}) [a]	Diffusion coefficient for ssDNA in solution and in hydrogel.
	R_{post}	100 (µm)	Radius of posts.
	d_{post}	310 (µm)	Center-to-center distance between posts.
Fig. 2	k_{1on}	6.5×10^2 (M^{-1}s^{-1}) [b]	Binding rate coefficient for P_1 + Activator and P_1 + Inhibitor.
	k_{2on}	3.2×10^2 (M^{-1}s^{-1}) [b]	Binding rate coefficient for P_2 + Inhibitor.
	k_{off}	1.2×10^{-2} (s^{-1}) [b]	Unbinding rate coefficient.
	$[P_1]_0$	25 (µM)	Initial concentration of P_1.
	$[\text{Activator}]_0$	2.24 (mM)	Initial concentration of Activator.
	$[\text{Inhibitor}]_0$	2.24 (mM)	Initial concentration of Inhibitor.
Fig. 3	k_{bi1}	5.5×10^5 (M^{-1}s^{-1}) [c]	Rate coefficient for Substrate + Catalyst.
	k_{bi2}	6.2×10^6 (M^{-1}s^{-1}) [c]	Rate coefficient for Intermediate 1 + Output.
	k_{uni}	4.2×10^{-2} (s^{-1}) [c]	Rate coefficient for Intermediate 1 → Catalyst + Waste 2.
	k_{leak}	7.4 (M^{-1}s^{-1}) [c]	Rate coefficient for Substrate + Fuel.
	k_{rep}	1.9×10^5 (M^{-1}s^{-1}) [c]	Rate coefficient for dqReporter + Output.
	$[\text{Catalyst}]_0$	1.0 (µM)	Initial concentration of Catalyst.
	$[\text{dqReporter}]_0$	1.0 (µM)	Initial concentration of dqReporter.
	$[\text{Substrate}]_0$	0.1 (µM)	Initial concentration of Substrate.
	$[\text{Fuel}]_0$	0.2 (µM)	Initial concentration of Fuel.
Fig. 4	k_{1on}	6.5×10^2 (M^{-1}s^{-1}) [b]	Binding rate coefficient for $P1_1$ + Input and $P1_1$ + Inhibitor.
	k_{2on}	3.2×10^2 (M^{-1}s^{-1}) [b]	Binding rate coefficient for $P2_1$ + Activator.
	k_{3on}	1.6×10^3 (M^{-1}s^{-1}) [b]	Binding rate coefficient for $P1_2$ + Inhibitor.
	k_{off}	1.2×10^{-2} (s^{-1}) [b]	Unbinding rate coefficient.
	$[P1_1]_0$	25 (µM)	Initial concentration of $P1_1$.
	$[P2_1]_0$	25 (µM)	Initial concentration of $P2_1$.
	$[\text{Input}]_0$	5.6 (µM)	Initial concentration of Input.

- Unless otherwise specified, initial concentrations of DNA species were set to 0 µM.
- The initial concentrations of species immobilized on the posts were set to half of the experimental values, as the post heights are half of the channel height, which corresponds to half the concentration in two dimensions.
- [a]Estimated by substituting the average length of diffusible ssDNA used in this study (46 nt) into an empirical formula $D = 4.9 \times 10^{-6}$ cm^{2}s^{-1} $\times$ [length (nt)]$^{-0.68}$ from earlier studies.[4,5]
- [b]From previous literature.[6]
- [c]From previous literature.[7]

4. Supporting Note 1

4.1 Parameter studies of negative-feedback-mediated communication

We performed reaction-diffusion simulations to examine how key parameters affect the collective spatiotemporal dynamics of the system. Following the analysis in Figs. 4 l-o, average concentrations and standard deviations of activated posts were calculated for inner, middle, and outer ones by averaging over 10 posts in each category (Figure S10).

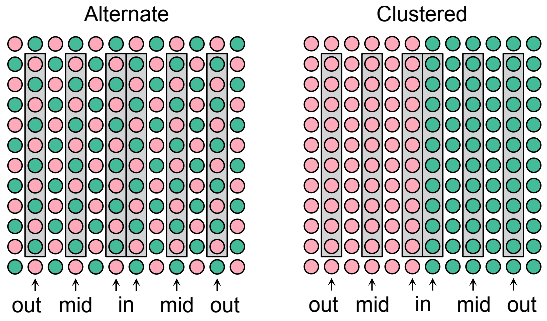

Figure S10. Posts for quantifying average concentrations at inner (in), middle (mid), and outer (out) positions in the post arrays.

4.1.1 Distance between posts

We varied the inter-post distances d relative to the default center-to-center distances d_{ref} = 310 µm (110 µm spacing) to 0.75 d_{ref} (33 µm spacing) and 1.25 d_{ref} (188 µm spacing). As shown in Figure S11 and Figure **S12**, shorter distances result in faster communication between posts, leading to faster macroscopic kinetics. In this regime, as is more prominent in the alternate arrangement, Posts 1 exhibit lower activation peaks, which can be attributed to more rapid quenching by the Inhibitor released from Posts 2. In contrast, larger inter-post distances lead to more homogeneous responses, as reflected by smaller standard deviations. When posts are more widely spaced, diffusing ssDNA can spread more freely in the surrounding space, which reduces local concentration gradients and attenuates spatial heterogeneity in the collective dynamics.

The clustered arrangement shows a pronounced earlier light up of outer Posts 1 (magenta) for smaller distances (Figure S12). This is due to the fact that the Input available within the array is insufficient to fully activate the posts, and additional diffusion from the surrounding needs to occur to activate all.

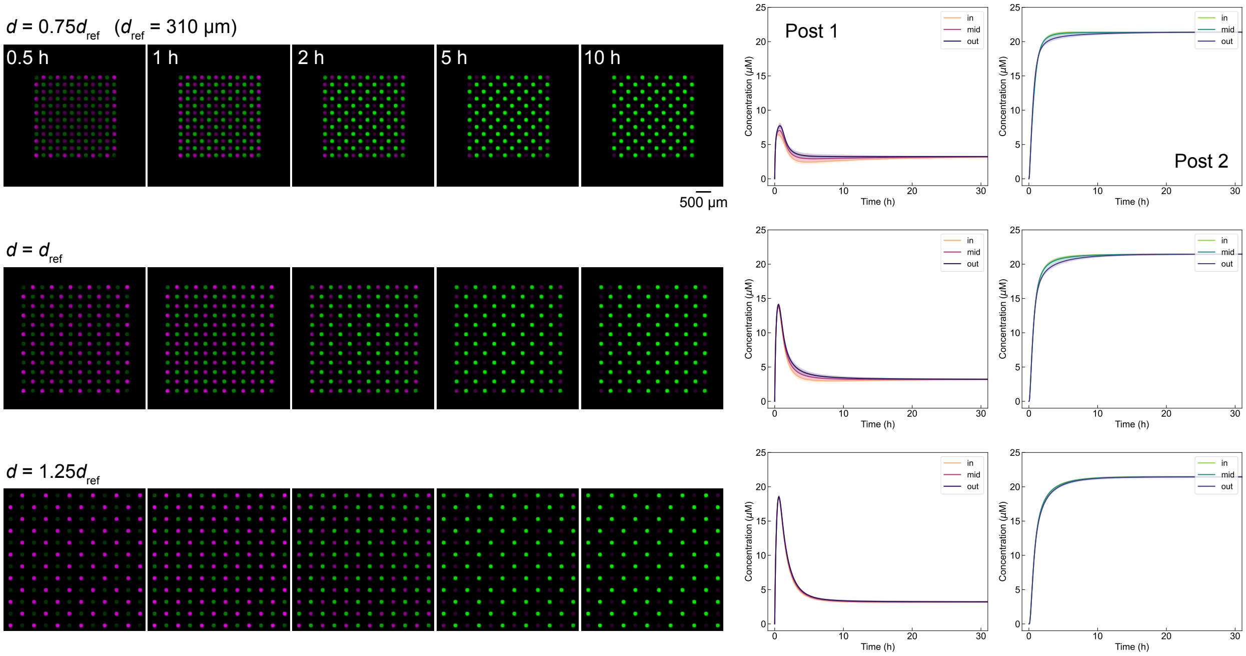

Figure S11. Influence of inter-post distance on collective spatiotemporal behaviors (alternate arrangement). Note that x-axes are cropped to 30 hours.

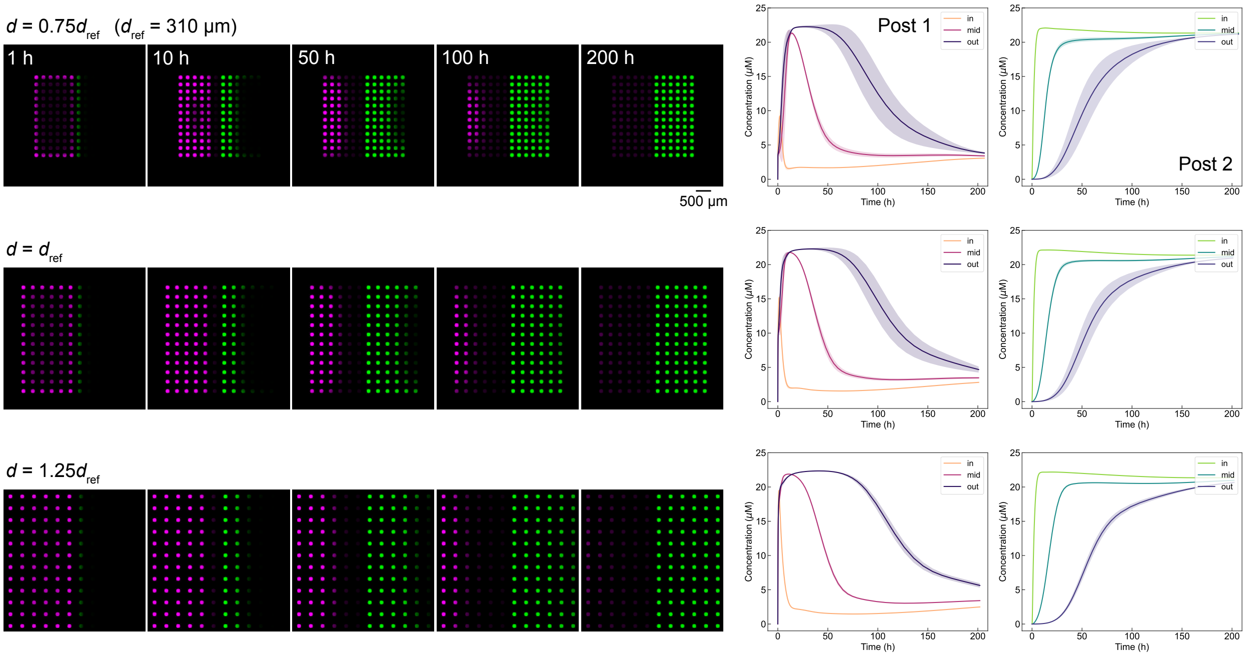

Figure S12. Influence of inter-post distance on collective spatiotemporal behaviors (clustered arrangement).

4.1.2 Diffusion coefficient of ssDNA in hydrogel posts

Diffusion of ssDNA in hydrogel posts can be experimentally tuned via the crosslink density of hydrogels, which controls the mesh size. In the main simulation, diffusion in the hydrogel posts was assumed to be comparable to that in the surrounding solution, and both regions were assigned the same diffusion coefficients D.

To investigate the influence of diffusion within posts, we reduced the diffusion coefficient in the post regions (D_g), relative to a reference value $D_{\mathrm{g,ref}} = 3.6 \times 10^{-7}$ cm^{2}s^{-1}, keeping the diffusion coefficient in the surrounding solution constant. As shown in Figure S13 and Figure **S14**, lower D_g leads to slower uptake and release of DNA within individual posts, visible as pronounced ring-like activation patterns. This local diffusion limitation propagates to the array scale, slowing inter-post communication and broadening the activation peaks of Posts 1 due to delayed activation and deactivation.

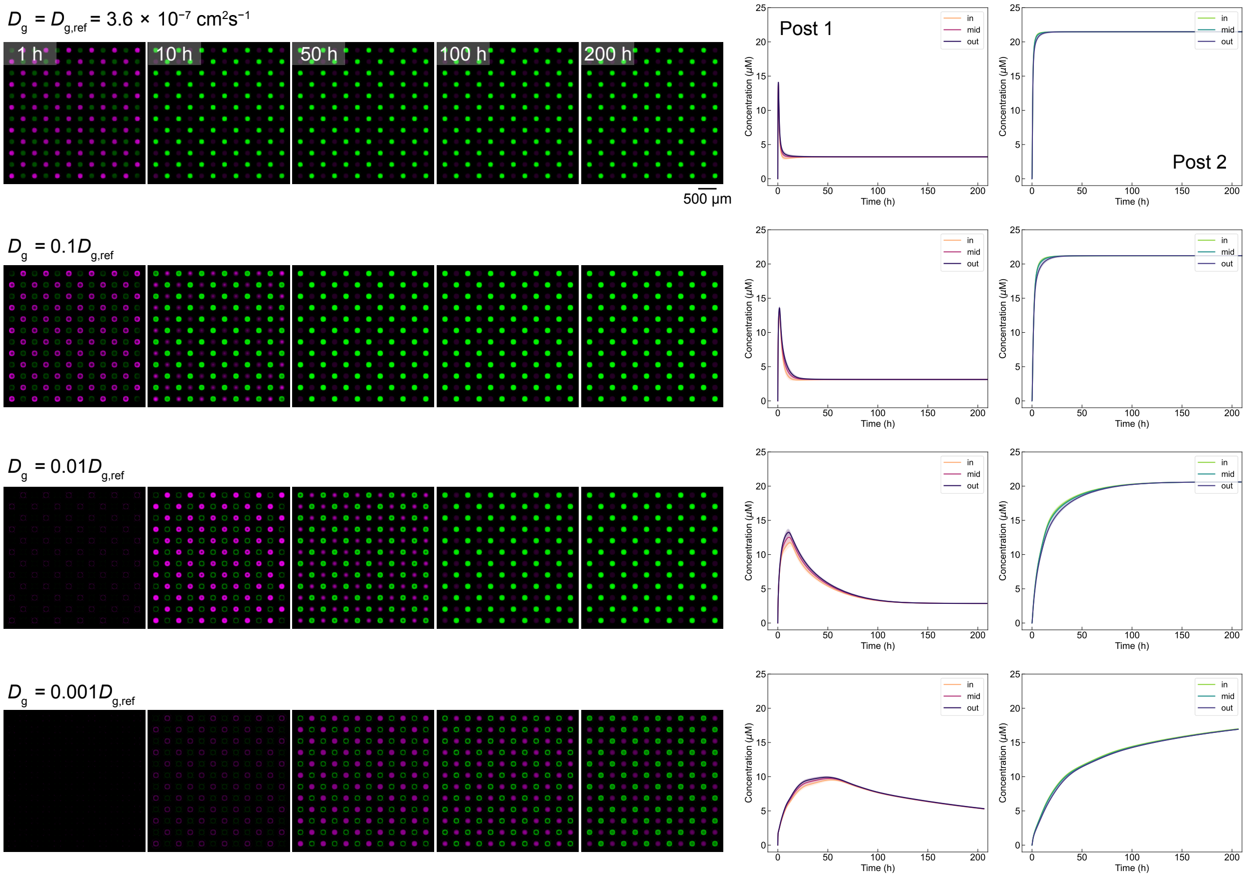

Figure S13. Influence of diffusion coefficients in posts on collective spatiotemporal behaviors (alternate arrangement).

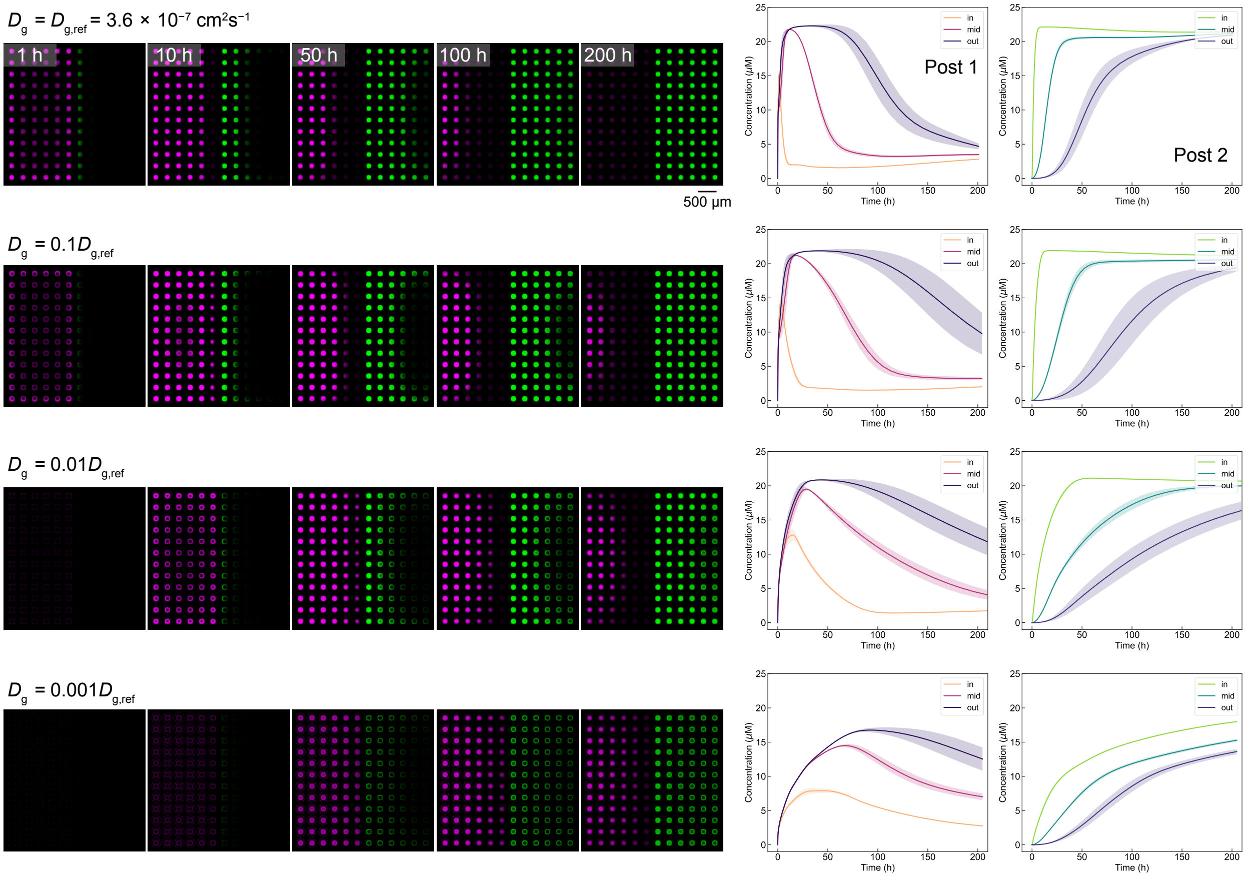

Figure S14. Influence of diffusion coefficients in posts on collective spatiotemporal behaviors (clustered arrangement).

4.1.3 Diffusion coefficient of ssDNA in both posts and solution

Unlike our artificial system, which is dominated by free diffusion in aqueous solution, diffusion of oligonucleotides inside living cells is known to be substantially slower (typically by one to two orders of magnitude) due to the highly viscous and crowded intracellular environment. To mimic such conditions, we uniformly reduced the diffusion coefficients (D) in both the hydrogel posts and the surrounding solutions from the reference diffusion coefficient $D_{\mathrm{ref}} = 3.6 \times 10^{-7}\ \mathrm{cm^2s^{-1}}$.

As seen in Figure S15 and Figure **S16**, decreasing the diffusion coefficient significantly slows the kinetics, more prominently than when only the intra-post diffusion coefficient D_{g} is reduced. This is because lowering diffusion in the solution phase directly retards long-range signal transmission between posts. Under strongly diffusion-constrained conditions in the clustered arrangement, outer Posts 1 become activated earlier than those located in the middle of the array. This behavior arises because, compared with the alternate arrangement, Posts 1 are more densely packed in the clustered arrangement, which enhances local depletion of Input within the array and creates a larger concentration gradient between the array interior and the surrounding solution. Under low-diffusion conditions, Input transport from the surrounding solution into the array becomes rate-limiting, preventing rapid relaxation of this gradient. As a result, outer posts, which are directly exposed to the solution, are activated earlier, while middle posts are activated with a delay.

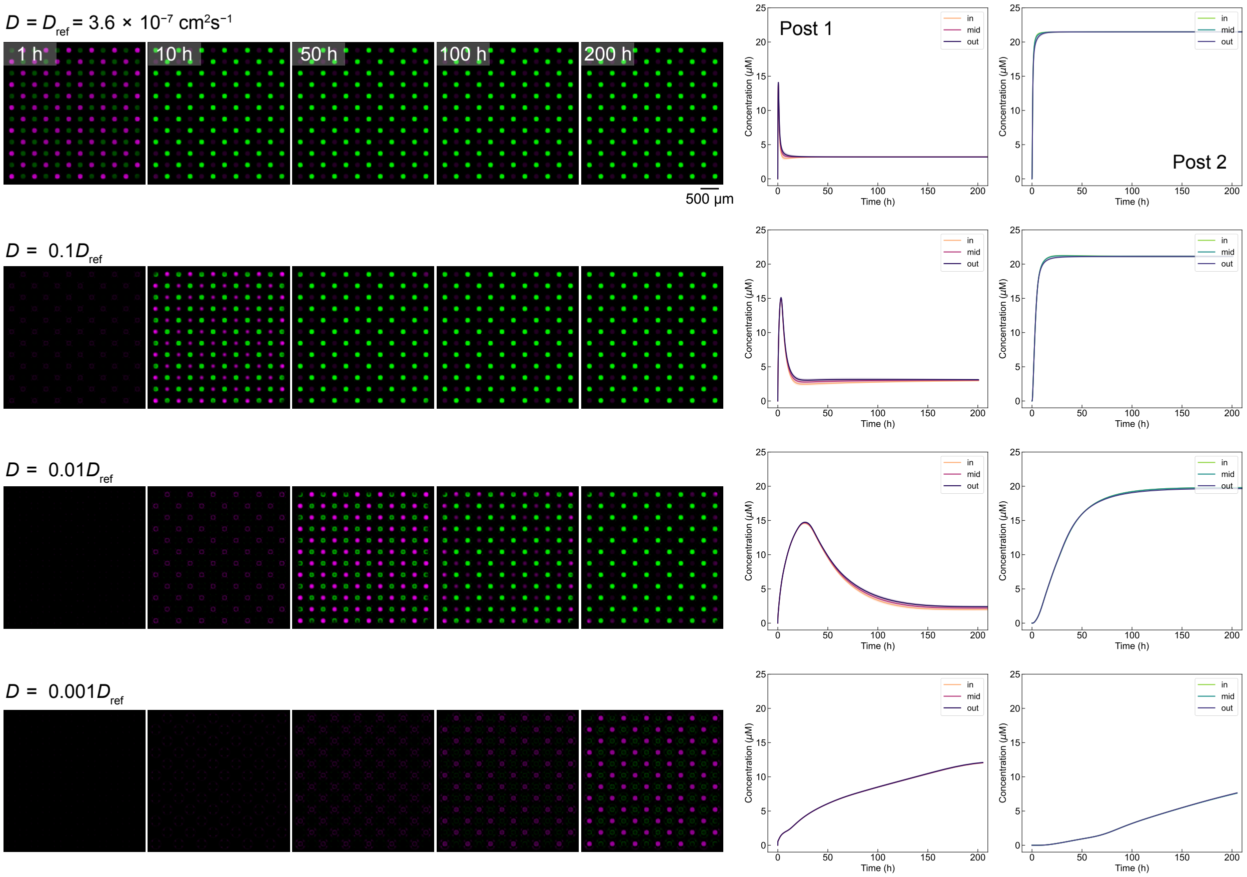

Figure S15. Influence of diffusion coefficients in posts and solutions on collective spatiotemporal behaviors (alternate arrangement).

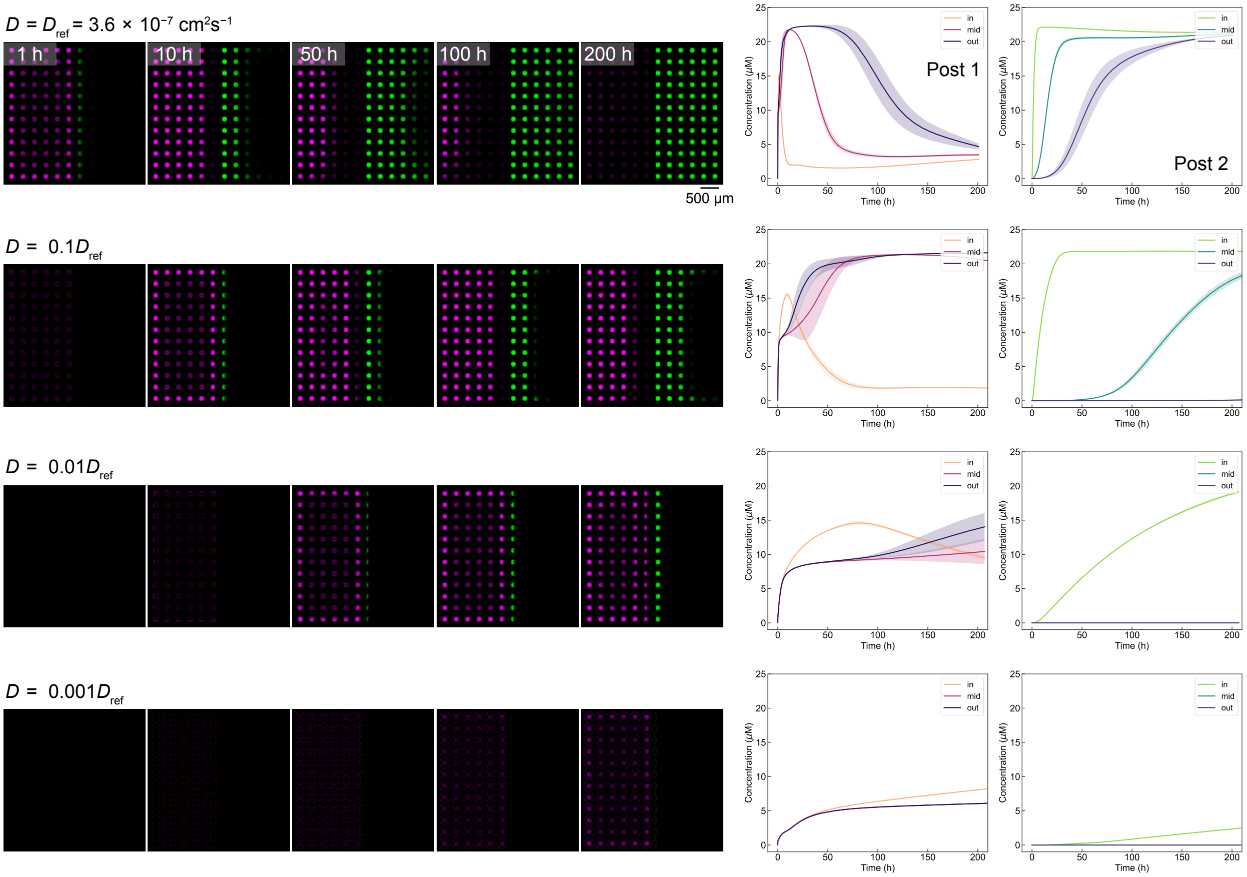

Figure S16. Influence of diffusion coefficients in posts and solutions on collective spatiotemporal behaviors (clustered arrangement).

4.1.4 Input concentration

Here, we varied the initial Input concentration $[\mathrm{Input}]_0$ relative to the reference value $[\mathrm{Input}]_{0,\mathrm{ref}} = 5.6$ µM.

Overall, higher $[\mathrm{Input}]_0$ leads to faster macroscopic kinetics (Figure S17 and Figure **S18**). The peak concentration of activated Posts 1 decreases as $[\mathrm{Input}]_0$ is lowered, an effect that is more pronounced in the alternate arrangement. In contrast, Posts 2 maintain high saturated concentrations even at low $[\mathrm{Input}]_0$. This asymmetry arises from the catalytic role of Input in the feedback loop. During a single negative-feedback cycle, activated Posts 1 release Input into the solution when they are quenched by Inhibitor (the third step in Fig. 4b). The released Input can be reused to activate remaining unreacted Posts 1, which in turn promote activation of Posts 2, while Posts 1 are subsequently quenched by a substantial amount of Inhibitor released from Posts 2. Because each activation of Posts 1 ultimately produces more Activator than the amount of initially added Input, Posts 2 can still be efficiently activated even when $[\mathrm{Input}]_0$ is low. In the clustered arrangement, the decrease in the peak level of activated Posts 1 is less pronounced. This is because Posts 2 gradually release Inhibitor from the interface with Posts 1, preventing a sudden quenching of all Posts 1. Meanwhile, Posts 1 release Input as they are gradually quenched, which can then slowly activate remaining unquenched Posts 1. At excessively high $[\mathrm{Input}]_0$, the large pool of free Input suppresses further net release of Input from Posts 1, thereby weakening the catalytic turnover within the feedback cycle.

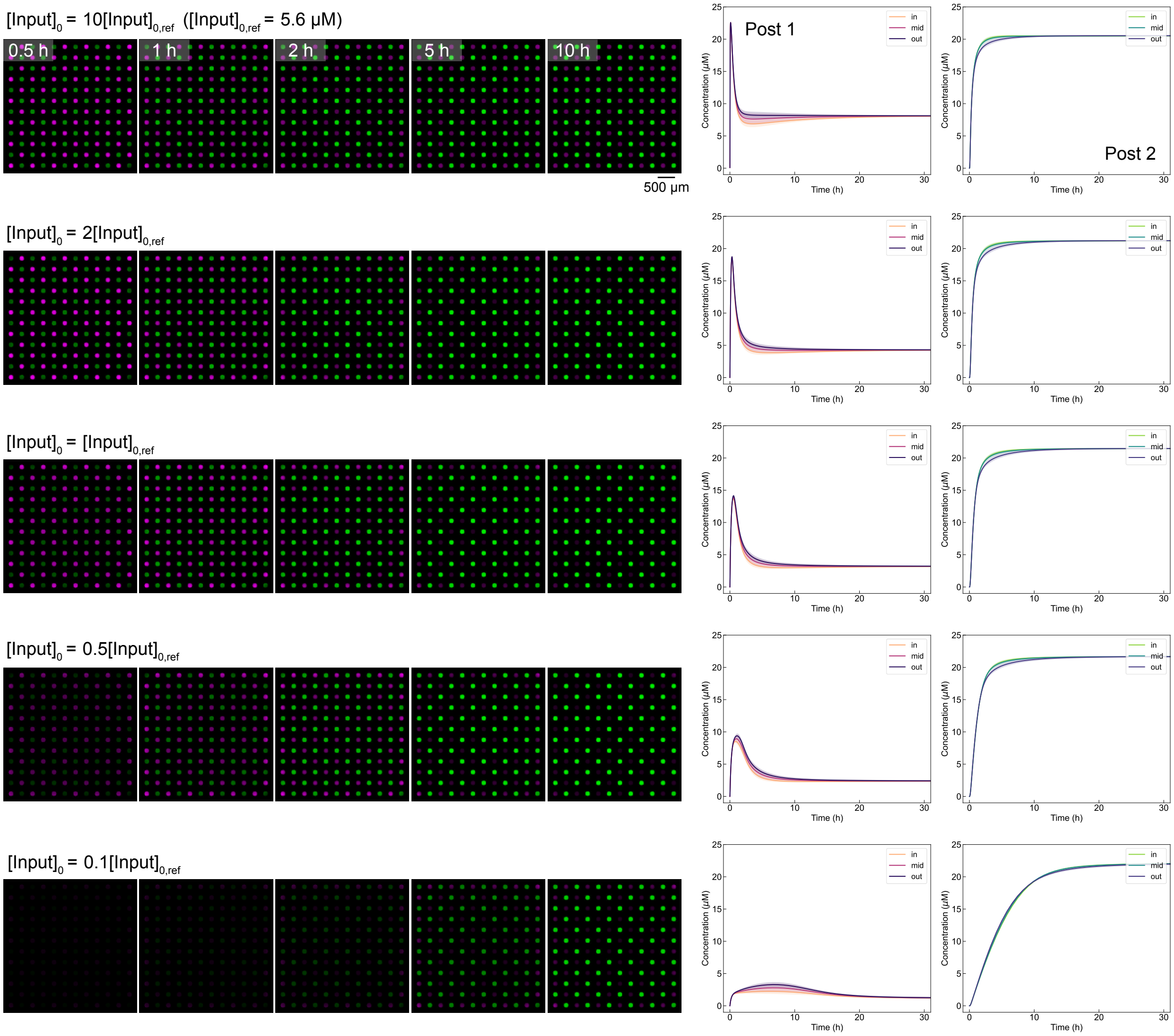

Figure S17. Influence of Input concentrations on collective spatiotemporal behaviors (alternate arrangement). Note that x-axes are cropped to 30 hours.

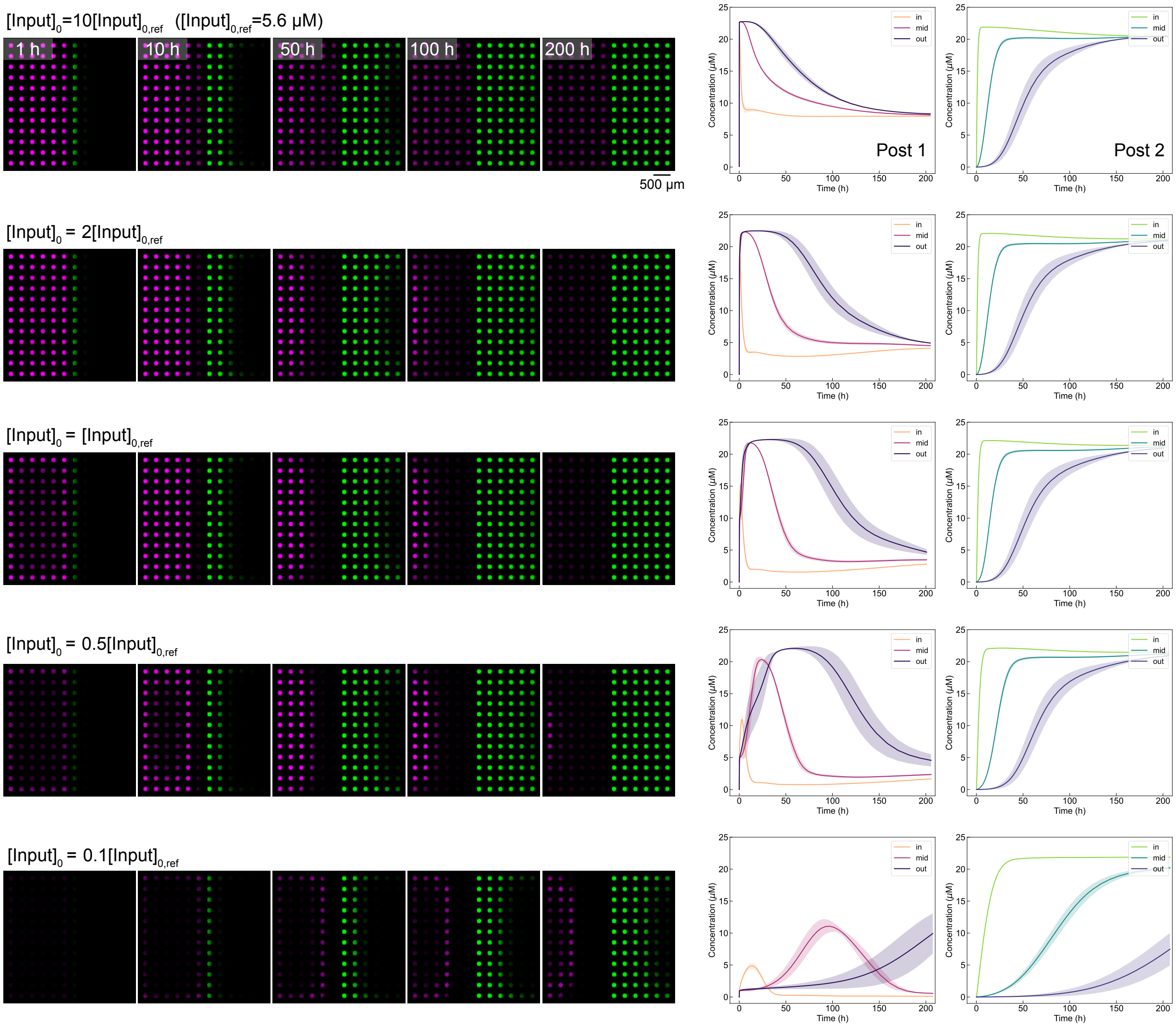

Figure S18. Influence of Input concentrations on collective spatiotemporal behaviors (clustered arrangement).

5. Supporting Videos

Supporting Video 1: Localized signals create distinct activation patterns.

Locally added activation/inhibition signals diffuse into the microfluidic channels and spatiotemporally compete for reactions with the hydrogel post arrays. This competition creates transient activation patterns that are unique by the input positions.

Supporting Video 2: Localized catalysts create spatially biased activation patterns.

Inter-post communication is achieved by implementing a catalytic signal generation. Separately patterned Catalyst posts locally produce Output from Substrate and Fuel, activating surrounding Reporter posts in a spatially biased manner.

Supporting Video 3: Arrangement determines communication efficiency.

A negative feedback loop enables more complex communication dynamics. Homogeneously added Input activates Posts 1 (magenta), which in turn activate Posts 2 (green); Posts 2 then inhibit Posts 1. The communication efficiency strongly depends on the spatial arrangements of posts. In alternate arrangements, both post types respond rapidly and synchronously, whereas in clustered arrangements, active communication is restricted to the interface between Posts 1 and 2.

References

(1) Phillips, R. J.; Deen, W. M.; Brady, J. F. Hindered Transport of Spherical Macromolecules in Fibrous Membranes and Gels. *AIChE J.* **1989**, *35*, 1761–1769. https://doi.org/10.1002/aic.690351102.

(2) Lira, L. M.; Martins, K. A.; Torresi, S. I. C. de. Structural Parameters of Polyacrylamide Hydrogels Obtained by the Equilibrium Swelling Theory. *Eur. Polym. J.* **2009**, *45*, 1232–1238. https://doi.org/10.1016/j.eurpolymj.2008.12.022.

(3) Sim, A. Y. L.; Lipfert, J.; Herschlag, D.; Doniach, S. Salt Dependence of the Radius of Gyration and Flexibility of Single-Stranded DNA in Solution Probed by Small-Angle x-Ray Scattering. *Phys. Rev. E* **2012**, *86*, 021901. https://doi.org/10.1103/PhysRevE.86.021901.

(4) Lukacs, G. L.; Haggie, P.; Seksek, O.; Lechardeur, D.; Freedman, N.; Verkman, A. S. Size-Dependent DNA Mobility in Cytoplasm and Nucleus. *J. Biol. Chem.* **2000**, *275*, 1625–1629. https://doi.org/10.1074/jbc.275.3.1625.

(5) Nkodo, A. E.; Garnier, J. M.; Tinland, B.; Ren, H.; Desruisseaux, C.; McCormick, L. C.; Drouin, G.; Slater, G. W. Diffusion Coefficient of DNA Molecules during Free Solution Electrophoresis. *Electrophoresis* **2001**, *22*, 2424–2432. https://doi.org/10.1002/1522-2683(200107)22:12<2424::AID-ELPS2424>3.0.CO;2-1.

(6) Groeer, S.; Schumann, K.; Loescher, S.; Walther, A. Molecular Communication Relays for Dynamic Cross-Regulation of Self-Sorting Fibrillar Self-Assemblies. *Sci. Adv.* **2021**, *7*, eabj5827. https://doi.org/10.1126/sciadv.abj5827.

(7) Zhang, D. Y.; Hariadi, R. F.; Choi, H. M. T.; Winfree, E. Integrating DNA Strand-Displacement Circuitry with DNA Tile Self-Assembly. *Nat. Commun.* **2013**, *4*, 1965. https://doi.org/10.1038/ncomms2965.
